# Separation of DNA Replication from the Assembly of Break-Competent Meiotic Chromosomes

**DOI:** 10.1371/journal.pgen.1002643

**Published:** 2012-05-17

**Authors:** Hannah G. Blitzblau, Clara S. Chan, Andreas Hochwagen, Stephen P. Bell

**Affiliations:** 1 Department of Biology, Howard Hughes Medical Institute, Massachusetts Institute of Technology, Cambridge, Massachusetts, United States of America; 2 Whitehead Institute for Biomedical Research, Nine Cambridge Center, Cambridge, Massachusetts, United States of America; 3 Department of Biology, New York University, New York, New York, United States of America; Stowers Institute for Medical Research, United States of America

## Abstract

The meiotic cell division reduces the chromosome number from diploid to haploid to form gametes for sexual reproduction. Although much progress has been made in understanding meiotic recombination and the two meiotic divisions, the processes leading up to recombination, including the prolonged pre-meiotic S phase (meiS) and the assembly of meiotic chromosome axes, remain poorly defined. We have used genome-wide approaches in *Saccharomyces cerevisiae* to measure the kinetics of pre-meiotic DNA replication and to investigate the interdependencies between replication and axis formation. We found that replication initiation was delayed for a large number of origins in meiS compared to mitosis and that meiotic cells were far more sensitive to replication inhibition, most likely due to the starvation conditions required for meiotic induction. Moreover, replication initiation was delayed even in the absence of chromosome axes, indicating replication timing is independent of the process of axis assembly. Finally, we found that cells were able to install axis components and initiate recombination on unreplicated DNA. Thus, although pre-meiotic DNA replication and meiotic chromosome axis formation occur concurrently, they are not strictly coupled. The functional separation of these processes reveals a modular method of building meiotic chromosomes and predicts that any crosstalk between these modules must occur through superimposed regulatory mechanisms.

## Author Summary

Sexually reproducing organisms rely on a specialized cell division called meiosis to produce genetically distinct gametes with half the chromosome number of the parent. The first stage of the meiotic cell division is the duplication of chromosomes, followed by the exchange of DNA between homologous chromosomes inherited from both parents. It has long been known that DNA replication occurs more slowly in pre-meiotic cells than in mitotically dividing cells, and it was postulated that this delay is due to the chromosome structures or proteins required for homologous DNA exchange. We show here that the delay of DNA replication in yeast is regulated separately from the formation of recombinant chromosomes; preventing recombination structures from forming does not alleviate the delays in pre-meiotic DNA replication, and cells lacking DNA replication are able to initiate recombination. We propose that these two processes are functionally separable and that the delay in pre-meiotic DNA replication in yeast may be a result of the starvation conditions required for the induction of meiosis in this organism.

## Introduction

The meiotic cell division produces haploid gametes from diploid progenitors by segregating the maternally- and paternally-derived copies of each chromosome. The faithful distribution of homologous chromosomes in meiosis is facilitated in most organisms by the crossovers formed during homologous recombination. Meiotic recombination occurs through the carefully orchestrated repair of programmed DNA double-strand breaks (DSBs) and takes place shortly after DNA replication during an extended gap phase referred to as meiotic prophase. Both the formation and faithful repair of meiotic DSBs into crossover recombinants requires the large-scale reorganization of each meiotic chromosome into a series of chromatin loops emanating from a central, condensed axis [1], [2]. Pre-meiotic S phase (meiS) is longer than pre-mitotic S phase (mitS) in many organisms [2], [3], [4], and it has been hypothesized that the protracted DNA synthesis either contributes to, or is affected by, the dramatic chromosome reorganization that occurs during meiotic prophase.

The kinetics of genome duplication are determined by where and when DNA replication begins. In eukaryotic genomes, DNA replication initiates from many sites along each chromosome, termed origins of replication, whose likelihood of utilization modulates the length of S phase in different developmental situations [5]. In yeast, potential replication origins are selected during G1 phase by the loading of the Mcm2-7 replicative helicase at specific sites along each chromosome 6,7. Upon entry into S phase, the activities of cyclin-dependent kinase (CDK) and Dbf4-dependent Cdc7 kinase (DDK) trigger the initiation of DNA replication from a subset of these potential origins [8], [9]. The remaining “inactive” origins are passively replicated by forks derived from nearby origins. Studies of individual DNA molecules revealed that the time at which each origin initiates DNA replication during S phase varies substantially between cells, and there is little correlation between distant loci, suggesting origin activation is not coordinated [10], [11]. Nevertheless, when the population as a whole is considered, a robust and reproducible replication timing program is seen, regardless of strain background or method used to assess replication timing [8], [9], [11], suggesting chromosomal DNA replication can be accurately described by a probability function.

MeiS in budding yeast has been estimated to last between 1.5–3 times as long as mitS [3], [12]. Theoretically, the longer duration of meiS could be due to either reduced efficiency of the initiation of DNA replication (from all or a subset of origins), reduced replication fork rates or a combination of both. Previous studies suggested that the extended length of meiS is not due to changes in origin selection because the majority of the origins on chromosomes III and VI initiate DNA replication during both mitS and meiS in budding yeast [13], [14], and genome-wide analyses suggested that origin selection is also similar in both S phases in fission yeast [15].

In budding yeast there is no clear separation of meiS and the start of prophase; DNA synthesis occurs concurrently with the loading of factors required for axis and DSB formation, and both require the same cell-cycle kinase activities. The meiosis-specific cohesin complex containing Rec8 is loaded onto chromosomes as cells enter meiS, and subsequently the axial proteins Hop1 and Red1 associate with the same axial core sites along each chromosome [16], [17]. As cells progress into prophase, chromosomes condense into a characteristic form, with a shortened axis and intervening DNA loops emanating away from the central core (reviewed in [2]). Association of both axial and DSB factors with core sites is critical for the formation of DSBs on the adjacent loops by the topoisomerase-like enzyme Spo11 [17], [18], whose proper association is dependent on Rec8 [16], [19]. A possible link between axis morphogenesis and S phase length was inferred from FACS analysis of total DNA content in yeast strains lacking Rec8 and Spo11 [12], and conversely, DNA replication timing has been implicated as a determinant of the time of DSB formation [20].

To better understand how the early meiotic cell division is coordinated, we characterized the kinetics and requirements of meiS and axis formation genome-wide in budding yeast. We found that origin firing was either delayed or less efficient at the majority of origins in meiS. Consistent with a decreased replication capacity, cells were more sensitive to nucleotide depletion during meiS. However, preventing meiotic chromosome reorganization had little effect on origin activation in meiS, suggesting that DNA replication is not strongly regulated by or linked to axis structure. Conversely, full DNA replication was not required for either axis assembly or DSB formation. Together, these data indicate that DNA replication and the initiation of homologous recombination are separable events, which coordinately contribute to the formation of meiotic recombinant chromosomes.

## Results

### Differential Mcm2-7 loading at a subset of pre-meiotic origins

**Figure 1:**
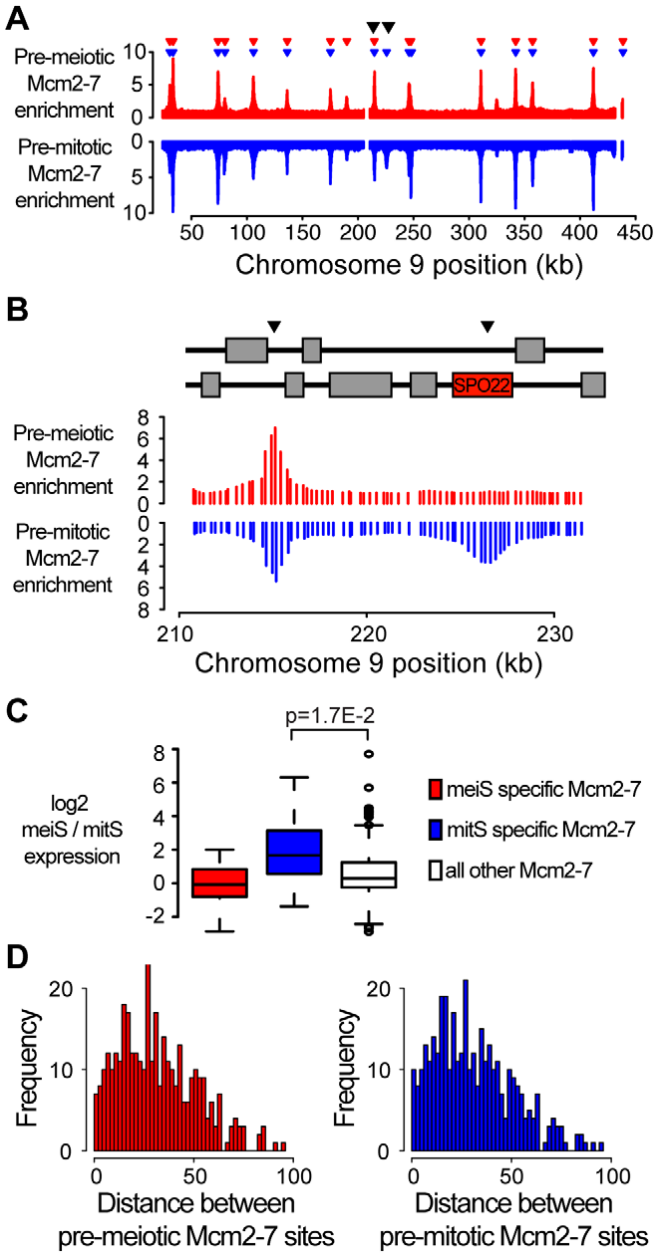
Transcription influences origin selection. (A) Mcm2-7 localization was performed for *cln3Δ* cells (A4224) prior to meiosis and a wild-type strain (SB1505) prior to mitosis. Mcm2-7 enrichment was plotted versus chromosome position for chromosome IX for meiotic cells (red, enrichment is upwards) and mitotic cells (blue, enrichment is downwards). Inverted red and blue triangles indicate significant Mcm2-7 binding sites. Black arrowheads indicate the positions of *ARS913* and the *SPO22* ARS. (B) As in (A), except a detailed view of *ARS913* (left) and the *SPO22* ARS (right) as indicated by arrowheads. The schematic above indicates the locations of coding regions. (C) Quantification of the change in gene expression for genes next to meiosis-specific (red), mitosis-specific (blue) and all other (white) Mcm2-7 binding sites. (D) Histogram showing the distribution of the calculated distances between Mcm2-7 binding sites prior to meiosis (red, left panel) and mitosis (blue, right panel).

To determine whether the initial selection of potential replication origins could explain the difference in S phase length between the meiS and mitS, we performed genome-wide location analysis for the Mcm2-7 helicase in pre-meiotic and pre-mitotic cells (Figure 1A and Figure S1). In total from both experiments, we observed Mcm2-7 binding at 393 loci, of which 382 had been identified previously as potential replication origins in mitotic cells in multiple strain backgrounds (Table S1). Comparison of the pre-meiotic and pre-mitotic Mcm2-7 binding sites revealed that the majority of origins loaded Mcm2-7 in both pre-meiotic and pre-mitotic cells (358/393, Table S1), consistent with the hypothesis that the mechanism of origin selection is the same in both cell cycles.

Although origin selection was conserved at most sites, we observed differential Mcm2-7 binding at 35 sites; 22 mitosis-specific and 13 meiosis-specific sites (Table S1 and Table S2). Sites where Mcm2-7 binding differed between pre-meiotic and pre-mitotic cells were more frequently located in coding regions or promoters than sites with similar Mcm2-7 binding in both cell cycles (20/35 versus 87/358, Chi-squared p = 8.1E-5). Origins are generally under-enriched in coding regions of the genome due to the incompatibility between transcription and replication factor binding [21]. Consistent with this incompatibility, mitosis-specific Mcm2-7 binding sites were found in sporulation-induced genes *SPO22* and *ZIP1*, and meiosis-specific binding sites were associated with mitotic budding-related genes *SHE2* and *BUD27* (Figure 1B and Table S2). Moreover, we observed significantly increased gene expression during meiS at mitosis-specific Mcm2-7 binding sites, compared to sites that bind Mcm2-7 in both cell cycles (Figure 1C compare blue and white boxes, t-test p = 1.7E-2), suggesting that differential Mcm2-7 binding at many of these sites was driven by changes in gene expression. Meiosis-specific Mcm2-7 binding sites did not show as clear a change in gene expression, suggesting that other mechanisms may also contribute to Mcm2-7 association. Nevertheless, the small number of changes in Mcm2-7 binding we observed are unlikely to account for the extended length of meiS, as we did not observe larger gaps between Mcm2-7 binding sites in meiotic cells (Figure 1D). Indeed, the meiosis- and mitosis-specific Mcm2-7 binding sites were consistently located close to other origins; the next Mcm2-7 binding site was on average 14 kb away with a maximum distance of 39 kb. In comparison, the average and maximum inter-origin distance for all potential origins was 30 kb and 95 kb, respectively (Figure 1D). These data indicate that the reduced rate of meiS is not due to differential origin selection.

### The same origins are active in the meiotic and mitotic cell divisions

**Figure 2:**
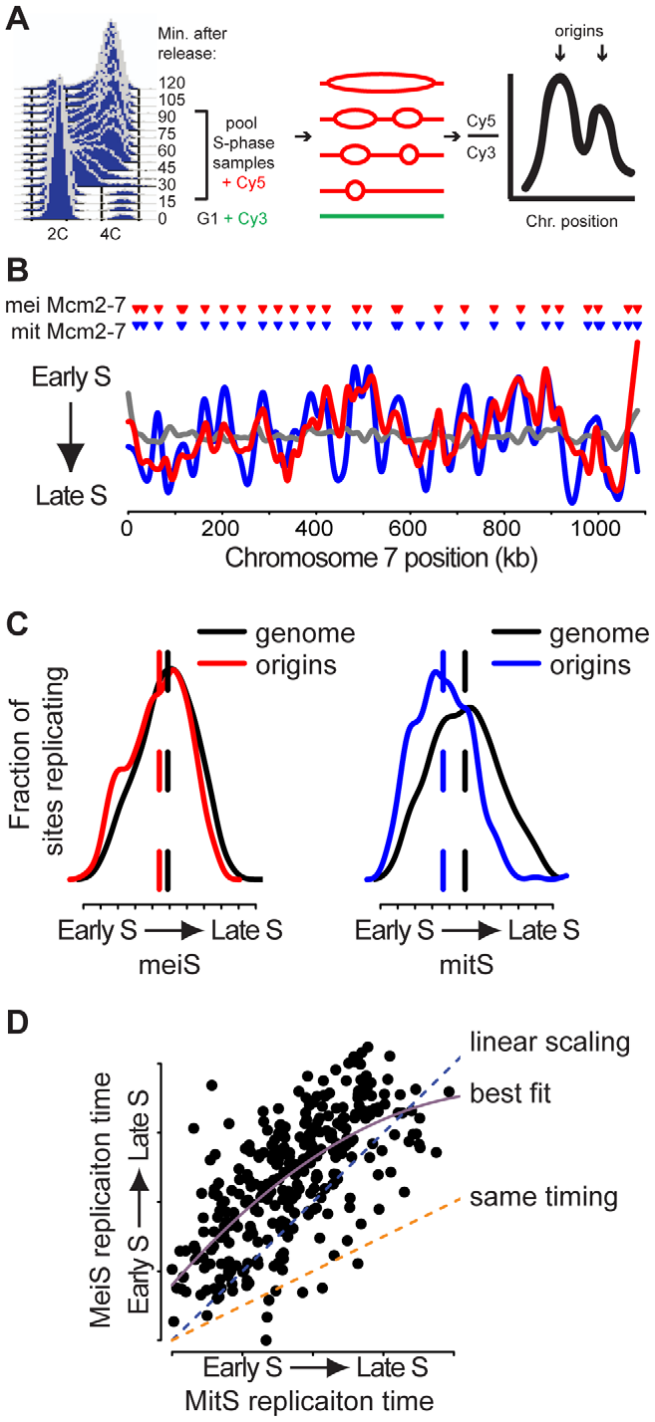
Meiotic DNA replication profiles. (A) *Ime2-as1-myc* homozygous diploid cells (KBY518) were synchronized in meiS. DNA samples were collected every 7.5 minutes. Resulting samples were pooled and co-hybridized with a G1 DNA sample to a tiled genomic microarray. (B) Replication profiles for meiS (KBY518, red line), mitS (SB1505, blue line) and control G1 vs. G1 (SB1505, grey line) hybridizations were created by plotting the smoothed log_2_ ratio (see Materials and Methods) versus chromosome VII position. Triangles indicate the positions of Mcm2-7 binding sites prior to meiosis (red) and mitosis (blue). (C) The distribution of relative replication time for all origins (colored lines) and for the entire genome (black lines) is plotted for meiS (left panel) and mitS phase (right panel). (D) The replication time in meiS of Mcm2-7 binding sites that were present in both cell cycles were plotted as a function of mitS replication time. Assuming meiS is twice as long as mitS, the orange dashed line indicates the predicted meiS replication time if origins replicated with the same kinetics in meiS and mitS. The blue dashed line is the predicted replication time trend line if scaling were linear with respect to S phase length. The purple solid line is the second order polynomial best-fit model.

Another possible explanation for the extended timing of meiS is that fewer sites with loaded Mcm2-7 complexes are used as origins of replication in meiS. To determine which potential replication origins were “active” during meiS, we measured the average replication time of sites across the genome in sporulating cells. To synchronize the cells as they passed through meiS, we used an ATP-analog-sensitive allele of the Ime2 kinase, *ime2-as1* [22]. Ime2 promotes meiotic cell cycle entry and pre-meiotic DNA replication [22], [23], so *ime2-as1* cells inoculated into sporulation medium containing the ATP-analog inhibitor for 4 hours did not initiate DNA replication (Figure 2A, 0 minutes). When the inhibitor was removed, cells progressed synchronously through meiS, as measured by FACS (Figure 2A). To determine the relative time of DNA replication, we pooled DNA samples that were collected every 7.5 minutes from the start to the end of meiS. The resulting samples were applied to a microarray together with a control (non-replicating) G1 sample to determine the relative abundance of DNA at 40,646 sites across the genome. Because the quantity of the DNA doubles when a site is replicated, sites that replicate earlier in S phase are enriched in the pool compared to sites that replicate later (Figure 2A) and the relative abundance of a given site in the S phase pool is inversely proportional to its average relative time of replication during S phase [9]. The results were visualized by plotting the relative enrichment in the S phase sample for all array features along a given chromosome, termed a replication timing profile (Figure 2B and Figure S2, red line). Peaks in the profile result from regions that replicated before neighboring sequences, and therefore must contain pre-meiotic origins of replication.

We next sought to compare the subset of origins that are active during meiS and mitS. We created pre-mitotic replication profiles for cells of the same genetic background (SK1) using alpha-factor synchronized cultures (Figure 2B and , blue line). The mitS replication profiles were very similar to those obtained in W303, another *S. cerevisiae* strain background (Figure S3), indicating that our method works similarly to those previously published and that the replication program is robust across different strains. To measure the level of random noise inherent in the copy number technique, we performed a control co-hybridization of two G1 DNA samples (Figure 2B and Figure S2, grey line). Because the smoothing algorithm used to create the profiles is unable to accurately predict replication time at the end of chromosomes, we excluded the 25 kb at each chromosome end from our analyses. After this exclusion, the meiS and mitS replication profiles showed a high degree of similarity (Pearson correlation coefficient = 0.64). Importantly, the peaks in both profiles were associated with sites of G1 Mcm2-7 binding (Figure 2B, inverted triangles), indicating that we could identify active origins of replication in both profiles. In general, the same peaks were present in both the pre-meiotic and pre-mitotic replication profiles, extending to all chromosomes the previous observations that the same origins initiate DNA replication during both meiS and mitS [13], [14]. Although we observed several instances in which meiosis- or mitosis-specific origins initiated replication (Figure S2), the effect on the overall profile was minimal, suggesting that these differences do not contribute substantially to S-phase progression.

### Replication initiation is delayed for many pre-meiotic origins

Although the majority of origins were active in meiS and mitS, examination of the replication profiles revealed that the relative replication time was different at many sites. The observation that many of the peak heights differed suggested that the timing or efficiency of replication initiation is altered at some origins in the meiotic cell cycle. When the distribution of replication timing for potential origins (Mcm2-7 binding sites) was compared to the whole genome in mitS, the majority of origins were replicated before bulk genomic DNA (Figure 2C, compare blue and black lines on right distribution). This finding is consistent with a model of efficient DNA replication initiation from these sites as cells enter mitS. Replication of potential origins was very limited at the end of S phase, consistent with this replication being comprised of fork progression and termination. In contrast, potential origin replication distributed more uniformly throughout meiS (Figure 2D, compare red and black lines), indicating that all origins did not initiate replication as efficiently upon entry into meiS.

To further investigate the delay in replication initiation in meiS, we compared the relative time of replication of all Mcm2-7 sites in mitS and meiS (Figure 2D). Given that there is no specific marker for the start and end of S phase in individual cells, or even the population as a whole, we used the FACS analysis to estimate the minimum length of meiS was 45 minutes at 30°C (Figure 2A), approximately twice as long as mitS [24]. If origins initiated replication at a similar time during meiS and mitS, we would expect the distribution of origin timing to resemble the orange dashed line (Figure 2D). If the time of replication were scaled with the length of S phase, but origins still fired in essentially the same order, we would expect the data to cluster around the blue linear-scaling prediction line (Figure 2D). We found that origins fired in a similar order in meiS and mitS, but 71% of potential origins replicated relatively later in meiS than mitS (Figure 2D, dots above the blue line). Consistently, we observed that 74% of origins replicated before the mean of the genome (mid-S phase) in mitS, but this number was reduced to 58% in meiS. We created a best fit model using linear regression (Figure 2D, solid purple line) [25], and found that the earlier origins were most delayed and the replication rate seemed to increase at the end of S phase, similar to predictions from other eukaryotes [26]. These data indicate that replication of many potential origins is delayed in meiS, either because of later initiation or passive replication in late S phase.

### Pre-meiotic cells have compromised replication capacity

**Figure 3:**
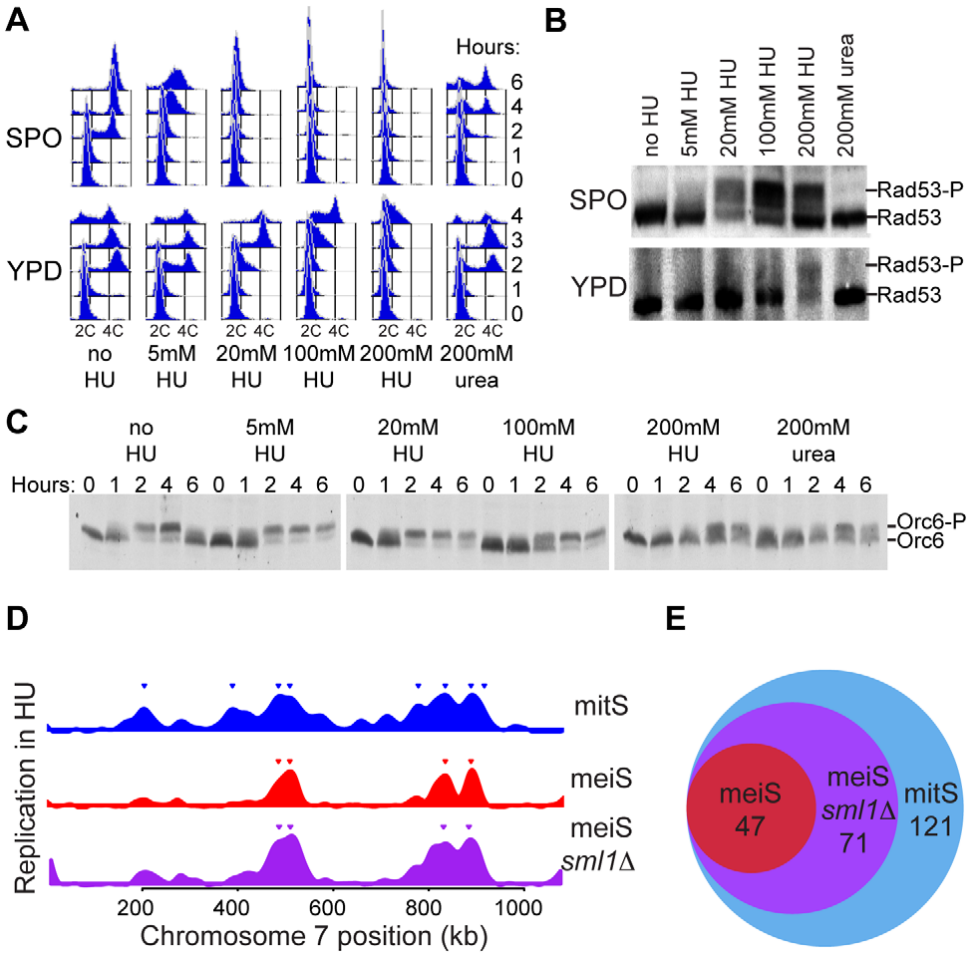
Reduced replication initiation in meiS. Pre-sporulation cultures of wild-type cells (H574) were inoculated into either SPO (top row) or YPD (bottom row) in the absence or presence of the indicated concentrations of HU or urea. (A) Comparison of response to HU in meiS and mitS as measured by FACS analysis. (B) Western blot analysis of Rad53 phosphorylation after 4 hours incubation in SPO (top panel) or YPD (bottom panel). (C) Western blot analysis of Orc6 phosphorylation in cells from the SPO cultures to monitor activation of CDK at the time of S phase entry. (D) The relative copy number enrichment of cells after 4 hours in 200 mM HU (mitS) or 20 mM HU (meiS) is plotted relative to chromosome VII position for wild-type (H574, blue for mitS and red for meiS) and *sml1Δ* cells (H4898, purple). (E) The total number of origins replicated in each of the conditions in (D) is represented as a Venn diagram.

To confirm the apparent differences in replication kinetics that we observed in the meiS and mitS replication profiles, we sought to identify all early replicating origins by delaying cells in early meiS with hydroxyurea (HU), as previously described for mitotic cells [9]. To begin, we tested a titration of HU concentrations to determine the optimal conditions to use with sporulating cells. We found that pre-meiotic cells were more sensitive to replication inhibition than mitotically dividing cells. First, the nature of the replication arrests differed. Pre-meiotic cells treated with 20–200 mM HU exhibited little or no replication progression, indicating that they had largely arrested replication in very early S phase (Figure 3A, SPO samples). In contrast, the mitotic cells inoculated into rich medium (YPD) containing 5–200 mM HU did not fully arrest DNA replication. Consistent with previous reports [27], even in the highest concentration of HU we observed detectible DNA replication after 2 hours (Figure 3A, YPD+200 mM HU). To further test the idea that cells are more sensitive to HU exposure in meiS, we measured the autophosphorylation associated with activation of the intra-S phase checkpoint kinase Rad53 by western blotting. Consistent with the idea that pre-meiotic cells are more sensitive to replication inhibition, Rad53 became hyper-phosphorylated at a much lower concentration of HU in meiS than mitS (Figure 3B).

Because it has been reported that cells sporulated in the presence of HU have reduced levels of early meiotic transcripts, including *IME2* [28], we were concerned that addition of HU may generally inhibit meiotic entry independent of DNA replication and S-phase checkpoint activation. To test whether cells had entered meiS in each of our experiments, we measured the CDK-dependent phosphorylation of Orc6 that occurs as cells enter S phase. We were able to detect Orc6 phosphorylation consistent with meiS entry in cells exposed to 5–200 mM HU, although the kinetics of Orc6 phosphorylation were delayed in the presence of high amounts of HU (Figure 3C). Addition of 200 mM urea, a similarly nitrogen-rich compound, also slowed meiS DNA replication and S-phase entry (Figure 3A, 3C). However, because this treatment did not inhibit mitS DNA replication (Figure 3A) or elicit a checkpoint response in meiS or mitS (Figure 3B), we conclude that high concentrations of HU or urea inhibit meiotic cell cycle entry independent of S-phase checkpoint activation. When HU- or urea-treated cells were released into sporulation medium without HU, they completed meiosis and formed viable spores, indicating they were reversibly inhibited and remained viable during the treatment (data not shown). Together, these data indicate that high concentrations of HU (or urea) can reversibly delay meiS entry. Therefore, we chose to use 20 mM HU for all further meiotic experiments, as this concentration of HU inhibited meiS progression without significant delays in meiotic cell cycle entry.

We used HU to determine the number and location of early-replicating origins in meiS and mitS. To create as similar a situation as possible, we synchronized cells in G1 in pre-sporulation medium, and subsequently divided the cultures into either sporulation medium (to induce meiosis) containing 20 mM HU or rich medium (to induce mitosis) containing 200 mM HU. After four hours, total DNA was collected and relative copy number was measured genome-wide. We detected replication initiation at a subset of sites in both pre-meiotic and pre-mitotic HU-arrested cells (Figure 3D, Figure S4 and Figure S5). The extent of replication in HU for each origin was similar to the time of replication of that site in the corresponding S-phase replication profile. The highest peaks in the HU profiles almost always coincided with the highest peaks in the corresponding replication timing curve (Figure S5). Thus, both the S phase and HU profiles detected the locations of early replicating origins.

We compared the identity of origins replicated in meiotic and mitotic cells exposed to HU. We considered all origins that showed copy number enrichment greater than half the maximum enrichment of the genome to be replicated in each HU experiment (Figure 3D and Figure S5, inverted triangles). Although the majority of these origins were associated with a clear peak in the HU profiles, indicative of active initiation, some of these origins also could have been passively replicated by the fork from a nearby origin. Consistent with previous results from mitotically dividing cells, we observed that all chromosomes contained multiple early-replicating origins in mitS. Most chromosomes contained early-replicating origins during meiS, although the sites on chromosomes VIII and XVI were just below the 50% cutoff in the meiS HU profile. Comparing the number of origins replicated in the meiS and mitS HU profiles revealed that many fewer origins initiated replication in HU in pre-meiotic cells (47 versus 121, Figure 3E and Table S1). All origins that were replicated in HU in pre-meiotic cells were also replicated in HU in pre-mitotic cells (Figure 3E), indicating that a subset of early mitotic origins also function efficiently in meiS, but that others become inhibited by HU during the sporulation program.

Given that meiotic cells were far more sensitive to inhibition of DNA replication by HU treatment (Figure 3A), we asked whether low nucleotide levels could explain the delayed replication initiation in meiS. We increased nucleotide levels by removing the ribonucleotide reductase (RNR) inhibitor *SML1* [29]. When we measured DNA replication in *sml1Δ* cells treated with HU, we found that increasing nucleotide levels increased the number of early origins to levels intermediate between meiS and mitS conditions (total of 71, Figure 3E and Figure S4). *SML1* deletion did not result in defects in meiotic S phase entry, sporulation efficiency or spore viability (data not shown). Given the sensitivity of meiotic cells to HU treatment, and the increases in DNA replication observed when nucleotide levels are increased, we propose that the starvation conditions required to initiate meiotic entry lead to low intra-cellular nucleotide levels that delay DNA replication.

### Centromeres are a strong determinant of early replication in meiS

**Figure 4:**
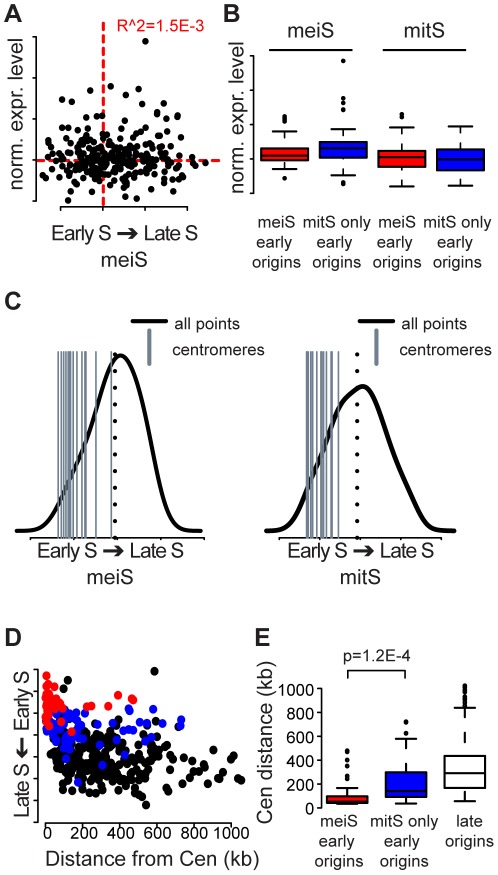
Centromeres replicate early in S phase. (A) The average expression level of origin proximal genes is plotted versus the time of replication in meiS. The red dotted lines indicate the population average. (B) The expression level distributions for meiS (left) and mitS (right) are plotted for the genes surrounding each origin for meiS early origins (red boxes) and mitS-only early origins (blue boxes). (C) The replication time for each centromere is indicated as a gray vertical bar compared to the distribution of replication time for the whole genome (black line) in meiS (left panel) and mitS (right panel). The mean replication time of the genome is indicated by the black dotted lines for each panel. (D) The replication time of each origin is plotted as a function of the distance of the origin from the closest centromere. MeiS early origins are indicated in red, mitS-only early origins are indicated in blue and late origins are colored black. (E) The data from (D) are summarized as box and whisker plots, with significance of the difference between mei-S and mitS-only early origins indicated.

In an attempt to explain the changes in replication initiation timing that occurred between meiS and mitS, we looked at the relationship between chromosomal features and replication timing. Because meiotic entry is associated with large changes in the gene expression program, we first explored the connection between replication timing and gene expression. Using published datasets, we determined the expression of all genes within 500 bp of meiS and mitS origins [30], [31]. We found no relationship between meiotic gene expression level and replication time in meiS (Figure 4A). We also examined expression of genes surrounding the 47 meiS early origins and the 74 mitS-only early origins that do not initiate replication in HU in meiS. We found there was no significant difference between the expression levels of genes adjacent to these two classes of origins (Figure 4B, compare red and blue boxes), again suggesting that the delay in meiS replication initiation is not due to transcriptional changes proximal to these origins. Similarly, we tested for a correlation between changes in time of replication and changes in gene expression between meiotic and mitotic cells, but found no relationship (data not shown), indicating that the large-scale changes in replication timing in meiS are independent of the meiotic gene expression program. Finally, we explored the locations of meiotic unannotated transcripts (MUTs) [32] and found no relationship between their presence and Mcm2-7 binding and origin activation (Table S2). For example, *ECM23/MUT1498* is predicted to cover *ARS1621*, yet we observed Mcm2-7 binding and a peak in the replication profiles indicating origin activation at this site in both meiS and mitS (Figure S2).

We next asked whether early replication of centromeres was conserved in meiS, because centromere proximal regions of chromosomes are replicated early during mitS in multiple yeasts [33], [34]. Indeed, we found that all centromeres replicated in the first half of S phase in both mitS and meiS, with an average replication time of 22% and 28% of S phase, respectively (Figure 4C). Additionally, centromere-proximal origins were highly enriched in the meiS HU profiles for every chromosome (Figure S4). Plotting the replication time of all origins during meiS as a function of distance from the centromere revealed that origins close to centromeres were consistently replicated earlier in S phase than origins farther from centromeres. Strikingly, meiS early origins were on average 70 kb from a centromere, and the majority (32 of 47) was within 50 kb of a centromere (Figure 4D red dots, Figure 4E red distribution). Conversely, the set of origins that are replicated early during mitS extended significantly further (average of 176 kb) from centromeres (Figure 4D, blue dots, Figure 4E, blue distribution, t-test p = 1.2E-4). The effect of centromere proximity on replication time extended 50–100 kb along the chromosomes, as the origins in this range replicated significantly earlier than those farther away (Figure 4C, 4D). The overall size of this 100–200 kb domain on each chromosome could, at least in part, explain why the smallest chromosome have a relatively high density of early origins and, on average, replicate early (Table S1 and Figure S2). These data reveal that, as in mitS, centromeres are a strong determinant of early meiS replication initiation, and the effect is more apparent during meiS due to compromised DNA replication capacity.

### MeiS timing does not correlate with meiotic chromosome structure

**Figure 5:**
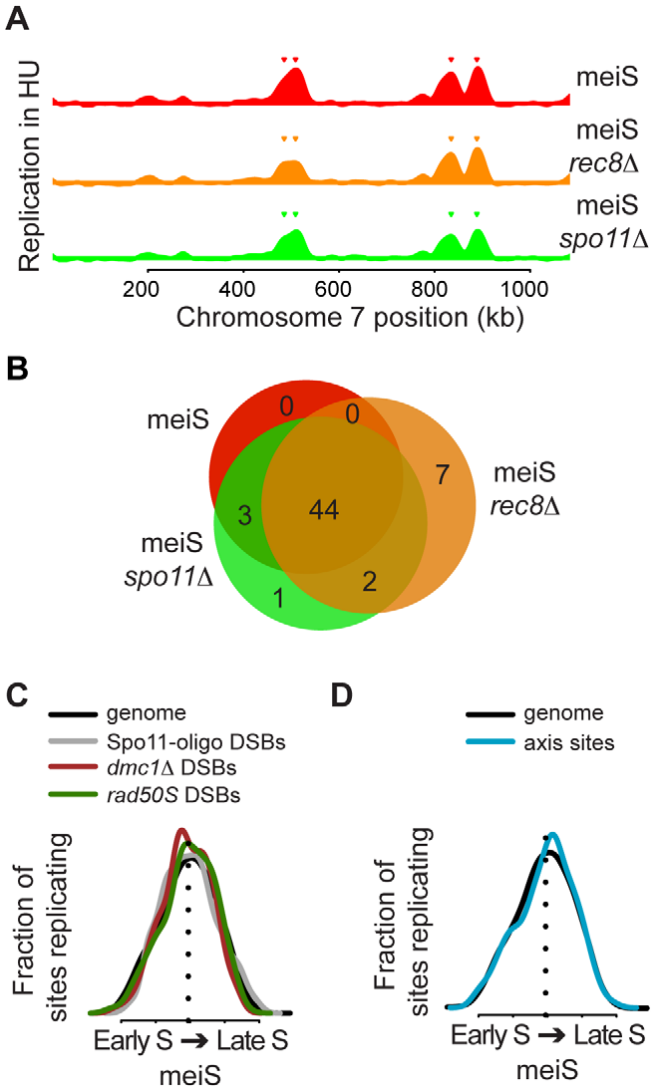
No relationship between DNA replication timing and recombination sites. (A) Chromosome VII replication profiles for pre-meiotic cells in the presence of 20 mM HU are shown for wild-type (H574, red), *rec8Δ* (H5187, orange) and *spo11Δ* (H5184, green) cells. Inverted triangles indicate the position of origins that are considered replicated in each strain. (B) Venn diagram summary of the experiment shown in (A), with the same color coding. (C) The distributions of replication timing in meiS are shown for the entire genome (black line), DSBs hotspots mapped by Spo11-oligo recovery (gray line), ssDNA enrichment in a *dmc1Δ* strain (brown line) and Spo11 binding in *rad50S* cells (green line). (D) The distributions of replication timing in meiS are shown for the entire genome (black line) and for axis association sites (blue line).

Given that meiotic chromosomes undergo large structural changes in preparation for recombination, and factors involved in these processes have been implicated in the control of meiotic replication, we investigated the relationship between pre-meiotic DNA replication and DSB formation. To understand whether axis or DSB formation delay meiS replication initiation, we measured early DNA replication (in the presence of HU) in cells unable to form meiotic axes (*rec8Δ*) or defective in DSB formation (*spo11Δ*). We observed similar HU replication profiles in sporulating wild-type, *rec8Δ* and *spo11Δ* cells: the vast majority of early meiS origins were replicated in all three strains (44 of 47 in wild-type cells, Figure 5A, 5B and Figure S4). These data indicate that Rec8 and Spo11 are not primarily responsible for the changes in meiotic replication origin timing that we observed.

We next determined the replication time of several chromosomal features during meiS, including DSB hotspots (HSs) and axis-associated core regions. Analysis of 3434 HSs mapped by Spo11-oligo accumulation [35] revealed that DSB sites were replicated throughout S phase (Figure 5C, grey line). When the HSs were ordered by rank, there was a slight trend that the stronger HSs were replicated earlier in S phase than the weaker sites, although the difference was not statistically significant (Figure S6A). We also measured the replication time of the strong DSB HSs mapped by either ssDNA enrichment in *dmc1Δ* cells or Spo11 genome-wide location analysis in *rad50S* cells [36] and found both were replicated with timing mirroring the entire genome (Figure 5C, brown and green lines, respectively, t-test p = 0.25 for *dmc1Δ* and p = 0.90 for Spo11 DSBs), indicating neither set of HSs are preferentially enriched in early or late replicating regions. Since many DSB factors associate with axis sites [17], we also measured the replication time of these regions. We defined axis association sites by overlapping localization of the axial proteins Rec8, Hop1 and Red1, which occurred at 565 sites in the genome (Figure S7, Table S3). As with HSs, axial sites were replicated throughout S phase, with a distribution similar to the whole genome (Figure 5D, t-test p = 0.97). Moreover, the change in timing of DSB and axis sites showed no trend toward earlier or later DNA replication (Figure S6B). The lack of detectable relationships between replication timing and the presence of axis and DSB sites suggests that meiotic chromosome structures do not strongly influence the timing of meiotic replication.

### DNA replication is not required for axial element association or DSB formation

**Figure 6:**
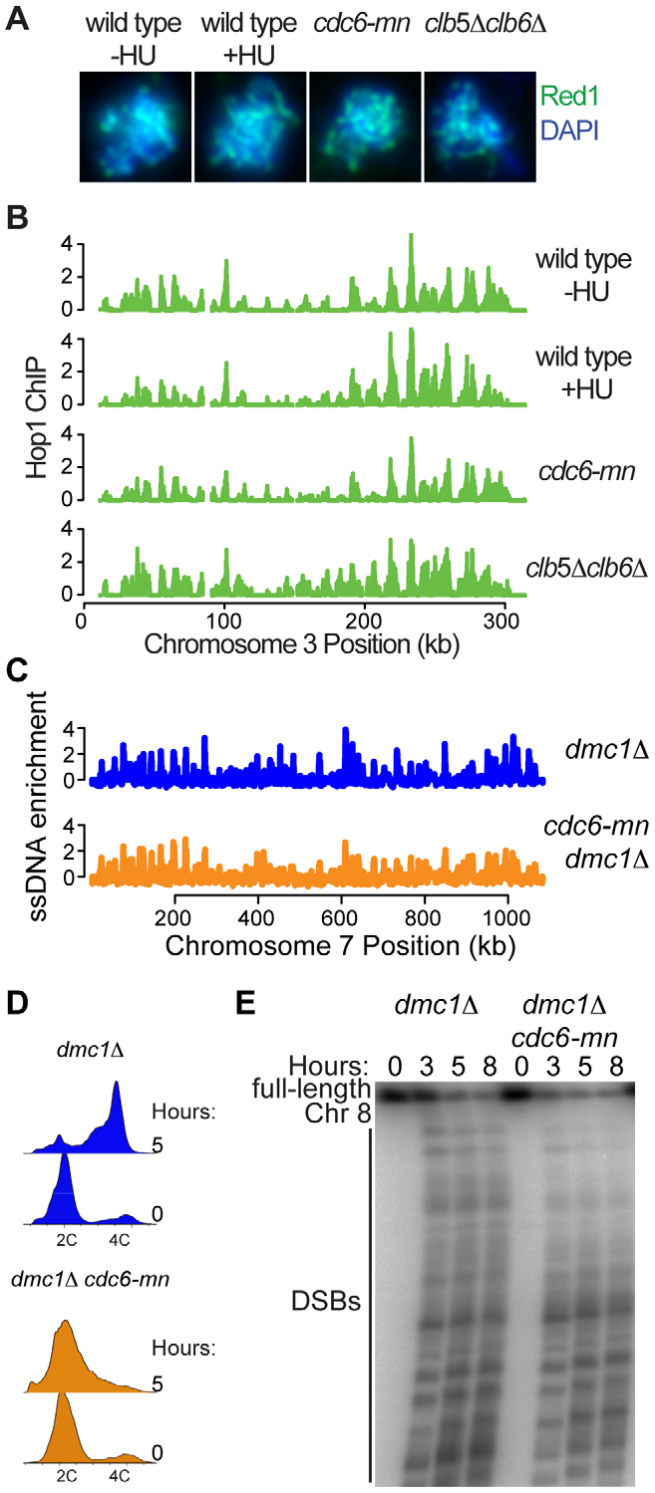
DNA replication is not required for axis association or DSB formation. (A) Indirect immunofluorescence for Red1 (green) and DAPI staining for total DNA (blue) on spread nuclei from cells at 3 hours after inoculation into SPO for wild-type cells (H119) with and without HU, *cdc6-mn* cells (H154) and *clb5Δ clb6Δ* cells (H2017). (B) Hop1 localization analysis was performed for wild-type cells with (H4471) and without HU (H119, [67]), *cdc6-mn* cells (H154) and *clb5Δ clb6Δ* cells (H2017). The enrichment of Hop1 over input DNA is plotted for chromosome III. (C) ssDNA enrichment in *dmc1Δ* cells (H118, [67]), and *dmc1Δ cdc6-mn* cells (H1584) was plotted with respect to position on chromosome VII. (D) FACS analysis was performed for *dmc1Δ* (H118) and *dmc1Δ cdc6-mn* cells (H1584). (E) CHEF gel analysis of chromosome VIII during a meiotic time course using *dmc1Δ* (H118) and *dmc1Δ cdc6-mn* cells (H4534).

Since axis formation was not a critical determinant of meiS replication timing, we wondered whether replication timing might instead contribute to axis formation. Therefore, we monitored axis formation by indirect immunofluorescence of the Hop1 and Red1 proteins on spread nuclei from cells lacking complete DNA replication. We inhibited DNA replication in 3 ways; by arresting cells in early S phase with HU, by depleting the Mcm2-7 loading factor Cdc6 (*cdc6-mn*), which severely decreases DNA replication, and by removing the cyclins Clb5 and Clb6, which prevents all pre-meiotic DNA replication [37]. In each case we observed Hop1 and Red1 distributed along chromosomes (Figure 6A for Red1, Hop1 data not shown), demonstrating that replication is not required for meiotic axis association. We noted that the chromosomes failed to condense and individualize in the *clb5Δ clb6Δ* nuclei, indicating that CDK activity and/or DNA replication are likely important for the full assembly of normal meiotic chromosome structures. However, previous analysis of Rec8 staining in *cdc6-mn* cells indicated that full DNA replication is not required to form full axes [38], [39]. To confirm that axis formation occurs on the same sites in the presence and absence of DNA replication, we localized Rec8, Hop1 and Red1 by whole-genome location analysis. As previously described, Hop1 (Figure 6B and Figure S7) and Red1 (Figure S7) localized to cohesin-associated regions (CARs) [17], [40], similar to both Scc1 in mitotic cells and Rec8 in meiotic cells [41]. Although the overall levels of binding varied, we found consistent patterns of Hop1, Red1 and Rec8 at CARs in all situations lacking DNA replication examined, indicating that axis formation occurs independently of DNA replication (Figure S7).

To determine whether the axes formed in these situations were functional, we measured genome-wide DSB formation by ssDNA enrichment in a *cdc6-mn* strain (a *dmc1Δ* mutation was used to prevent repair of DSBs). We were able to detect DSBs across all chromosomes after 5 hours in sporulation medium (Figure 6C and Figure S8), despite the fact that the genome remained largely unreplicated at this time (Figure 6D). These DSB HSs occurred at the same sites in both *dmc1Δ* and *cdc6-mn dmc1Δ* cells, although the intensity of DSB formation differed at many sites. Because the FACS analysis indicated that there is some DNA replication occurring in the *cdc6-mn* strains (Figure 6D, see tailing towards 4C at 5 hours), we were concerned that the ssDNA at DNA replication forks might interfere with the quantitative measurement of DSBs in the *cdc6-mn* cells. Therefore, we measured DSB formation by pulsed-field gel electrophoresis, revealing high levels of DSBs in the *cdc6-mn* strains (Figure 6E, note that the total signal is lower in the *cdc6-mn* samples because we normalized for cell number and the chromosomes do not replicate). We conclude that the formation of DSB-competent meiotic chromosomes does not require bulk meiotic DNA replication. Together, our results indicate that pre-meiotic DNA replication and meiotic chromosome axis assembly are functionally separable processes, and that the formation of a fully DSB-competent chromosome configuration can occur in a chromosome-autonomous fashion without the need for a sister chromatid.

## Discussion

We investigated the coordination between pre-meiotic DNA replication and the formation of meiotic chromosome axes. Comparison of replication profiles from meiotic and mitotic cells revealed substantial differences in the regulation of initiation of DNA replication at many origins. Because the majority of replication origins initiated replication later in pre-meiotic cells, we propose that the slower meiS is primarily due to delayed replication initiation. We did not observe a direct link between the formation of DSB-competent chromosome structures and the DNA replication program, indicating that these processes can be functionally separated.

### Transcription regulates Mcm2-7 loading at a subset of origins

Although the Mcm2-7 binding sites were largely the same in both the mitotic and meiotic cell cycles, approximately 9% of sites showed differential Mcm2-7 loading. These sites were much more frequently located within promoters or coding regions of genes, and Mcm2-7 loading appeared to be prevented by gene expression. Previous reports indicated that transcription through an origin is deleterious to replication complex assembly and replication initiation [21], [42]. Similar to the situation described here, it has been reported that *ARS605* is inactivated by meiosis-specific transcription of the overlapping gene *MSH4*, which caused the loss of ORC-DNA association [14]. We did not identify *ARS605* as a mitosis-specific origin in this study (Figure S1), possibly because we collected samples for Mcm2-7 analysis relatively early in the meiotic cell cycle, when *MSH4* transcription was not yet fully activated and residual amounts of Mcm2-7 were still bound to the DNA. Alternatively, the low levels of Mcm2-7 we detected at *ARS605* are insufficient for initiation. However, we identified additional origins, at which Mcm2-7 association was similarly regulated by transcription of the locus. We note that none of the meiosis-specific origins identified in this study were novel; either ARS activity or Mcm2-7 binding was detected in previous studies using mitotically dividing cells (http://www.oridb.org). However, none of them were shown to initiate DNA replication in genome-wide mitotic studies, suggesting they do not function in their chromosomal context. Because these transcriptionally-regulated origins were located close to other origins, their inactivation is unlikely to have a substantial effect on the completion of DNA replication.

### Delayed DNA replication during meiS

Given that origin selection and activation were highly similar in meiS and mitS, the reduced rate of meiS must be due to delayed replication initiation or slow fork progression rates. The presence of a greater amount of noise in the meiS replication profiles made it impossible to measure relative fork rates in meiS and mitS, as we could not create an algorithm to specifically locate and measure all fork progression regions. Despite this difficulty, we noted that in some regions of the genome the scaled meiS and mitS profiles had similar slopes (Figure S2). Because the time of meiS is approximately twice the length of mitS and the profiles are scaled to S phase length, a similar slope indicates that the meiotic fork rates are approximately half the rate of the mitotic forks. On the other hand, we found strong evidence for a delay in replication initiation, as the majority of origins replicated later in meiS than mitS. This replication delay could be due to later or reduced efficiency of initiation, which the CGH method does not distinguish. In support of delayed initiation, a much smaller number of meiS origins initiated replication in HU. Although previous studies observed a similar efficiency of origin usage in meiS and mitS [13], [14], we found that the small chromosomes monitored previously showed smaller delays in meiS replication than larger chromosomes (Figure S2), leaving open the possibility that many origins exhibit a reduced efficiency of initiation in meiS. Recent studies suggest that timing and efficiency are linked [43], [44], so it is possible that both contribute to the delayed replication in meiS.

We postulate that the reduced replication initiation in meiS is caused by a limiting initiation factor. One candidate is the level of CDK activity, which is required for replication initiation. Removal of the cyclin Clb5 in mitotic cells causes a reduction in late origin activation, but the presence of Clb6/CDK substitutes to drive mitS [45], [46]. In meiotic cells, *CLB5* deletion has an even more severe phenotype, with very delayed and inefficient replication [37], suggesting residual Clb6/CDK activity is lower in meiotic cells. It seems less likely that DDK activity is limiting in meiS, as the use of an analog-sensitive allele of Cdc7 revealed that pre-meiotic DNA replication is virtually unaffected in the presence of the inhibitor [47]. It has been proposed that DDK must act at each individual origin at the time of initiation of DNA replication [48], [49], and it is possible that because replication initiation is spread out over a much longer time period in meiS, less DDK activity is required to support these lower initiation rates. A second and non-exclusive model is that slow pre-meiotic DNA replication is caused by the reduced dNTP levels in meiS [50], which presumably occur because of the starvation conditions used to induce sporulation. Lowered nucleotide levels could account for a decrease in both initiation and fork progression rates. This hypothesis is reminiscent of studies of mitotic growth in the presence of HU, which also causes both a delay in origin activation and slower fork rates, resulting in a protracted S phase [27]. The observation that meiotic cells arrest more tightly in response to HU is also consistent with a model of reduced dNTP levels.

Why is replication initiation delayed in meiS? The generalized delay in replication initiation observed in meiS is very similar to the scaling of S phase observed in other mutants that slow DNA replication [25], suggesting that cells respond to S phase challenges by decreasing the number of active forks. Although a slow S phase would be detrimental in competitive mitotic cultures because it would decrease growth rate, the meiotic program is a form of terminal differentiation and cells are not prepared to divide again immediately following meiotic exit. In sporulating cells, it may be advantageous to proceed slowly but accurately through the cell cycle. It has recently been shown that excessive replication initiation leads to genome instability [43], and meiotic errors would be propagated by the progeny. In higher eukaryotes, meiosis takes place only in germ cells, which differentiate within special organs in response to specific developmental cues. Although these germ cells are not limited by nutrient availability, they may also be optimized for fidelity.

### Early replication of centromere-proximal origins

It is interesting to note that not all origins were equally delayed during meiS; centromere-proximal origins initiated replication efficiently during both meiS (This study and [13]). The conservation of early replication timing of centromeres in meiS and mitS (reviewed in [33], as well as in distantly related yeast species [34], suggests that these sites play a critical role in determining the replication timing program. Consistent with this idea, it has been observed that moving a centromere was sufficient to change replication timing of adjacent origin sequences [51]. One hypothesis for early centromere replication is that it is important for kinetochore function, because mutants that change replication timing were shown to interfere with chromosome segregation [52]. Given that every chromosome has a centromere, it is also possible that linking early replication to centromeres helps to guarantee that every chromosome initiates replication in S phase, even when replication is compromised.

### Separation of pre-meiotic DNA replication from the assembly of break-competent meiotic chromosomes

Although it was previously observed that Rec8 and Spo11 regulate the length of meiS [12], our findings indicated that the activation of the earliest replicating origins is not directly regulated by these proteins. Deletion of either Rec8 or Spo11 did not significantly alter the profile of early pre-meiotic replication in HU. Similarly, DSB sites and chromosome core sites were distributed at random with respect to replication time in meiS, indicating no direct link between replication delays and either axis assembly or DSB formation. Therefore, we suggest that meiosis-specific cohesion and the Spo11 protein are not responsible for the altered replication timing program that we observed in meiS.

Similarly, full DNA replication is not required for the formation of break-competent meiotic chromosomes. The association of the axial proteins Hop1 and Red1 can be detected in HU-treated, *cdc6-mn* and *clb5Δclb6Δ* cells, all of which fail to duplicate their genomes, indicating that the formation of chromosome axes does not require either DNA replication or the presence of a connected sister chromatid. Association of Hop1 and Red1 occurs at cohesin-associated regions on chromosomes and is regulated by Rec8. It has been shown that mitotic and meiotic cohesin complexes load onto chromosomes independent of DNA replication [19], [38], [39], [53], and we propose that axial proteins are similarly able to load onto unreplicated chromosomes. We have also shown that DNA replication is not required for DSB formation, as full levels of DSBs form in *cdc6-mn* cells, which complete very little DNA replication (Figure 6), extending our previous finding that *cdc6-mn* cells form DSBs at the engineered *his4-LEU2* hotspot [54]. It has been observed that Spo11 is first loaded onto centromeres in meiS, before it becomes redistributed to sites along chromosome arms [19], and it is possible that the early replication of centromeres drives Spo11 association. However, we were unable to detect the specific replication of centromeres in *cdc6-mn* cells by CGH analysis (data not shown), yet they were able to form full levels of DSBs, indicating that Spo11 loading does not require early centromere replication.

Although the wild-type kinetics of DSB formation in *cdc6-mn* cells indicates that chromosome axis assembly and DSB formation proceed on a timer that does not require DNA replication, it is clear that locally delaying DNA replication by eliminating origins on one chromosome arm retards proximal DSB formation [20], [55]. The data presented here indicate that DSB formation is not intrinsically dependent on replication fork passage. The apparent discrepancy of these results could be explained if the single, severely delayed replication fork on the origin-less chromosome arm were to locally disrupt the loading or phosphorylation of DSB factors, delaying the initiation of DSB formation. In this case, the depletion of replication forks in the *cdc6-mn* cells would allow DSB formation. Alternatively, the processes could be coupled through a checkpoint mechanism triggered by the single delayed replication fork. In mitotic cells, the intra-S phase checkpoint down-regulates the activity of DDK [56], [57]. As it was demonstrated that DSB formation requires higher levels of DDK activity than DNA replication [47], the intra-S phase checkpoint could prevent DSB formation in wild-type meiotic cells, but may be lacking in *cdc6-mn* cells due to the severe decrease in DNA replication, similar to results obtained in *S. pombe* [58], [59]. In either model, DNA replication is not a prerequisite for DSB formation, but rather the processes would be coordinated by superimposed regulatory mechanisms.

Whereas much effort has been made to understand the events of meiotic prophase, relatively little is known about the regulation of meiS and the assembly of specialized chromosome structures necessary for meiotic recombination. In many organisms, the signals that initiate meiosis are unclear, and meiotic cell cycle entry cannot be determined by molecular or cytological markers until after the initiation of recombination. Studies in *S. pombe* revealed that pre-meiotic DNA replication initiates from the same sites as mitotic DNA replication, but replication is delayed or less efficient for a significant number of origins [15]. Our results indicate that this delay is due to limiting replication capacity, and reveal that centromeres are a strong determinant of early replication timing. Additionally, in *S. pombe* DNA replication is also not required for DSB formation, as multiple mutants that decrease DNA replication form DSBs across all chromosomes [58]. Therefore, the modular regulation of DNA replication and meiotic chromosome formation is conserved across distantly related yeast species, and could extend to other organisms as well.

## Materials and Methods

### Strains and growth conditions

Strains used in this study are isogenic to SK1 and are listed in Table S4. Gene disruptions were carried out using a PCR-based protocol [60]. *FLO8* was deleted in SB1505 to reduce flocculation. Cells lacking *CLN3* were used for Mcm2-7 Genome-wide location analysis experiments to increase efficiency of meiotic entry. To induce synchronous meiosis, strains were pre-inoculated at OD_600_ = 0.2 in YPA (1% yeast extract, 2% bactopeptone, 1% potassium acetate, Figure 1 and Figure 2), or BYTA medium (1% yeast extract, 2% tryptone, 1% potassium acetate, 50 mM potassium phthalate, Figure 3, Figure 4, Figure 5, Figure 6), grown for 16 hours at 30°C, washed twice with water, and resuspended at OD_600_ = 1.9 in SPO medium (0.3% potassium acetate, pH 7.0) at 30°C. For G1 control DNA, mitotic Mcm2-7 chromatin immunoprecipitation and mitS phase profiles, cells were inoculated into fresh YPD medium containing 5 µg/ml alpha-factor for 3 hours. For mitS profiles, cells were released into S phase by washing with 2 volumes of sterile water and resuspended in YPD at 30°C. MeiS cells containing the *ime2-as1* allele were synchronized by incubation in SPO+20 µM 1-NA-PP1 (Toronto Research Chemicals) for 4 hours, the cells were washed with 2 volumes of sterile water and resuspended in SPO medium. Samples of 1.5 mls were removed every 5 minutes for mitS profiles and every 7.5 minutes for meiS profiles. All DNA samples inclusive of S phase (those which showed cells in S phase in the FACS profiles, as well as two time points before and after) were pooled for processing. For hydroxyurea (HU) experiments, cells were inoculated into YPD containing 200 mM HU or SPO containing 20 mM HU for 4 hours at 30°C.

### Chromatin immunoprecipitation

25 mls of cells were harvested after 1 hour (Mcm2-7) or 3 hours (Rec8, Hop1, Red1) in SPO. For pre-mitotic Mcm2-7 analysis, 50 mls of culture at OD_600_ = 0.8 were collected from alpha-factor arrested cultures. Chromatin immunoprecipitation (ChIP) was performed as described [61]. One tenth of the lysate was removed as an input sample. Samples were immunoprecipitated for 16 hours at 4 degrees with UM185 (Rabbit polyclonal anti-Mcm2-7, 2 µl serum used per immunoprecipitation), 3F10 (Rec8-3HA, Roche, used 2 µg per immunoprecipitation), anti-Hop1 or anti-Red1 (N. Hollingsworth, 2 µl each serum used per immunoprecipitation).

### FACS analysis

Cell pellets from 100 µl of sporulation culture were fixed in 70% ethanol overnight at 4°C. Ethanol was removed and cells were resuspended in 500 µl of 50 mM sodium citrate (pH 7.0) containing 20 µg RNaseA for 16 hours at 50°C. Subsequently, 100 µg of Proteinase K were added and samples were incubated an additional 24 hours. 500 µl of 50 mM sodium citrate (pH 7.0) containing 2 µM Sytox green (Invitrogen) were added. Cells were sonicated briefly on lowest power and scanned using a FACSCalibur (BD Biosciences).

### DNA extractions for CGH

Cells were lysed by bead beating in 500 µl phenol/chloroform and 500 µl of breakage buffer (10 mM TRIS, pH 8.0, 1 mM EDTA, 100 mM NaCl, 2% Triton X-100, 1% SDS). After centrifugation, the aqueous phase was precipitated with ethanol and resuspended in 500 µl TE7.6 (10 mM TRIS, pH 7.6, 1 mM EDTA) with 30 µg RNase. DNA was resuspended and samples were incubated at 50°C for 3 hours. DNA was sheared by sonicating at 100% output and lowest power for 10 seconds using a Branson sonicator. DNA was extracted with 500 µl phenol/chloroform, precipitated with ethanol and resuspended in 100 µl of TE7.6

### Fluorescent labeling and microarray hybridization

For ChIP experiments, one half of the immunoprecipitated DNA and one tenth of the input DNA were labeled. For pooled S-phase and HU replication profiles, ∼5 µg of DNA from replicating or G1-arrested cells were labeled. Samples were labeled with Cy3-dUTP and Cy5-dUTP by random priming using 4 µg random nonamer oligo (IDT) and 10 units of Klenow (New England Biolabs, Beverly, MA). Unincorporated dye was removed using microcon columns (30-kDa MW cutoff, Millipore, Bedford, MA), and samples were co-hybridized to custom Agilent arrays (Wilmington, DE) using a standard protocol.

### Microarray data analysis

For each co-hybridization, Cy3 and Cy5 levels were calculated using Agilent Feature Extractor CGH software. Background normalization, log_2_ ratios for each experiment and scale normalizations across each set of triplicate experiments were calculated using the sma package [62] in R, a computer language and environment for statistical computing (v2.1.0, http://www.r-project.org). The raw data and log ratios analyzed in this study are available from the NCBI Gene Expression Omnibus (http://www.ncbi.nlm.nih.gov/geo/), accession number GSE35667.

Mcm2-7 binding sites were defined as sites that were significantly enriched (P<0.05) in 3 independent experiments. Array features within 500 bp of each other on the chromosome were merged into a single binding site. Mcm2-7 binding sites were assigned to a previously characterized origin if they overlapped the defined region (http://www.oridb.org). Clear Mcm2-7 peaks were detected at some sites that did not make the statistical cutoff. They were manually included in the list of binding sites if they corresponded to a known origin and an Mcm2-7 binding site was called in the other data set (meiotic or mitotic). For analysis of replication timing, Mcm2-7 sites were defined as the ACS, when one was predicted [63] or previously defined (http://www.oridb.org), or else as the midpoint of the minimally defined origin region. The replication time of each Mcm2-7 binding site was determined for each experiment by assigning it to the time of the closest point on the smoothed and predicted replication timing curve. Points <25 kb from chromosome ends were excluded from timing analysis due to the inability to accurately predict timing at chromosome ends.

For pooled S-phase and HU DNA samples, DNA replication profiles were smoothed and predicted every 50 bp using the loess smoothing spline with a span = 0.025 and a spar = 0.45 (Table S5). Mcm2-7 binding sites were considered replicated in HU if their value was greater than half of the maximum value in the genome (excluding points <25 kb from chromosome ends).

Axis association sites were defined as those with overlapping Rec8, Hop1 and Red1 binding at more that one adjacent chromosomal feature, defined similarly to Mcm2-7 binding sites, except with P<0.15. The positions of axis association sites were taken to be the midpoint of the intersection of the binding peaks of all three proteins.

Analysis of the replication time for axis association sites, Spo11 binding sites and ssDNA-enriched sites was performed as for the Mcm2-7 binding sites.

### Gene expression analysis

To measure gene expression, we analyzed the average of expression values at 15 and 20 minutes post alpha-factor from the mitotic data set of Granovskaia at al. [30] and the average of the 2- and 3-hour expression data for wild-type cells from Borde et al. [31]. The expression data sets were scale-normalized to a mean log value of 0 and a standard deviation of 1 over the 4987 genes for which data were available in both sets. Origin-proximal transcripts are those within 500 base pairs of the Mcm2-7 binding site. We repeated all analyses using the dataset of Primig et al. [64] and Friedlander, et al. [65], and obtained highly similar results (data not shown).

### Indirect immunofluorescence on spread nuclei

Meiotic nuclear spreads were performed as described [66]. In brief, the nuclei of spheroplasted cells were spread on a glass slide in the presence of paraformaldehyde fixative and 1% lipsol. After drying, the slides were blocked in blocking buffer (0.2% gelatin, 0.5% BSA in PBS) and stained with anti-Red1 (N. Hollingsworth, 1∶250 dilution).

### Analysis of ssDNA enrichment

The genome-wide analysis of DSBs in the *cdc6-mn* strain using ssDNA enrichment was conducted as described [36].

### CHEF and Southern analysis

Clamped-homogeneous electric field (CHEF) gel electrophoresis and Southern blotting were performed as described [36].

## Supporting Information

Figure S1 Mcm2-7 localization. Mcm2-7 enrichment was plotted versus chromosome position for all 16 chromosomes for meiotic cells (red, enrichment is upwards) and mitotic cells (blue, enrichment is downwards). Inverted triangles represent the significant binding sites we identified. White dots indicate the position of the centromere on each chromosome.
(TIF)

Figure S2 MeiS and mitS replication profiles. The smoothed, predicted replication timing profiles are shown for each of the 16 chromosomes for meiS (red lines), mitS (blue lines) and G1 vs. G1 control hybridization (grey lines). Mcm2-7 binding sites are indicated by inverted triangles for meiotic (red) and mitotic (blue) cells. Black dots indicate the positions of centromeres.
(TIF)

Figure S3 Comparison of mitS replication profiles for 2 yeast strains. MitS replication profiles for all chromosomes are shown for the current study in SK1 (blue lines) and for W303 (green lines) from Yabuki and colleagues [9]. Black dots indicate the positions of centromeres.
(TIF)

Figure S4 HU replication profiles. The extent of replication after 4 hours in HU is plotted for all 16 chromosomes for wild-type cells (H574) in YPD+200 mM HU (blue lines), SPO+20 mM HU for wild-type cells (H574, red lines), *sml1Δ* cells (H4898, purple lines), *rec8Δ* (H5187, orange lines) and *spo11Δ* (H5184, green lines). Inverted arrowheads denote origins that are replicated in each strain of the corresponding color. Black dots indicate the positions of centromeres.
(TIF)

Figure S5 Comparison of timing and HU profiles. The extent of replication after 4 hours in HU is plotted for all 16 chromosomes for wild-type cells (H574) in YPD+200 mM HU (blue histogram) and SPO+20 mM HU for wild-type cells (H574, red histogram). Above each histogram the corresponding S phase replication timing profile is plotted in blue for mitS and red for meiS. Black dots indicate the positions of centromeres.
(TIF)

Figure S6 DSB hotspot replication time. (A) The distribution of relative replication time for HSs mapped by Spo11-oligo recovery are shown for all 3434 non-telomeric HSs (grey box), and the 3434 HSs ranked in quintiles from lowest break number (purple) to highest break number (red). The timing distribution for the whole genome is shown as a white box. (B) The meiS replication time of DSB HSs mapped by ssDNA enrichment (left panel) or Spo11 genome-wide location analysis (center panel), or axis association sites (right panel) are plotted as a function of their time of replication in mitS. Red line indicates the predicted trend if relative replication time were identical in meiS and mitS.
(TIF)

Figure S7 Axis sites. Genome-wide localization analysis was performed for Rec8 (shown in purple), in wild-type cells with and without HU (H4471) *cdc6-mn* cells (H5491) and *clb5Δ clb6Δ* cells (H6495). Hop1 localization analysis is shown in green for wild-type cells without HU (H119, [67]), with HU (H4471), *cdc6-mn* cells (H154) and *clb5Δ clb6Δ* cells (H2017). Red1 localization analysis is shown in red for wild-type cells without HU (H119), with HU (H4471), *cdc6-mn* cells (H154) and *clb5Δ clb6Δ* cells (H2017). The enrichment of immunoprecipitated over input DNA is plotted for chromosome III. Sites that showed significant coincident binding for Rec8, Hop1 and Red1 are indicated by inverted black triangles above the plots. Black dots indicate the positions of the centromere.
(TIF)

Figure S8 ssDNA enrichment profiles. The ssDNA enrichment profiles for *dmc1Δ* cells (blue, [67]), and *dmc1Δ cdc6-mn* cells (H1584, orange, enrichment downwards) were plotted with respect to position for all 16 yeast chromosomes. Black dots indicate the positions of centromeres.
(TIF)

Table S1 Mcm2-7 binding sites identified in this study.
(XLS)

Table S2 Mitosis- and meiosis-specific Mcm2-7 binding sites.
(XLS)

Table S3 Axis sites identified in this study.
(XLS)

Table S4 Strains used in this study.
(DOC)

Table S5 Smoothed predicted profiles used in this study.
(XLS)
