# Supplementary figures and images for: Separation of DNA Replication from the Assembly of Break-Competent Meiotic Chromosomes
Source: PLoS Genet. 2012 May 17;8(5):e1002643. doi: 10.1371/journal.pgen.1002643 (PMC3355065; doi:10.1371/journal.pgen.1002643)

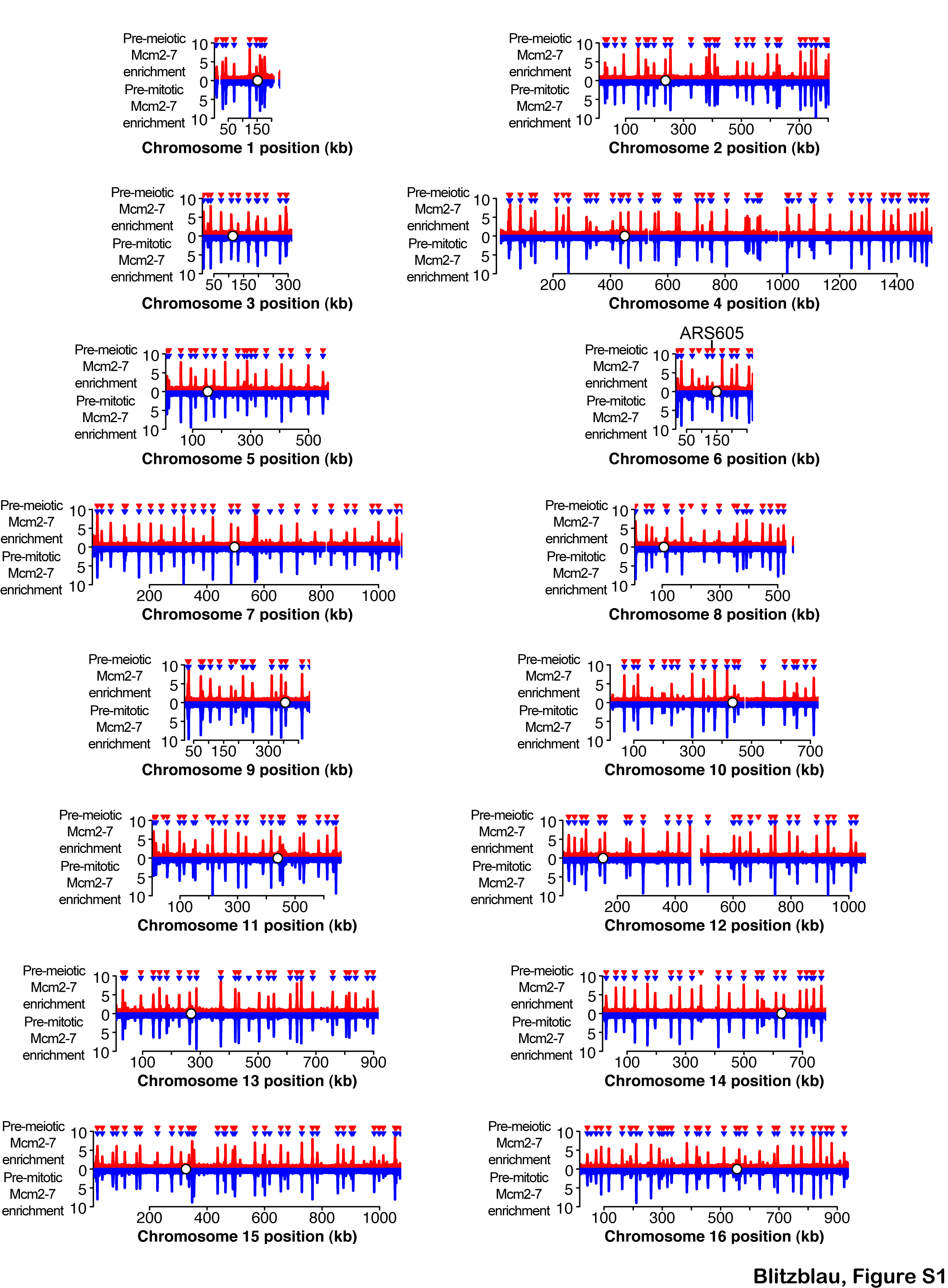

Supplement: Figure S1 — Mcm2-7 localization. Mcm2-7 enrichment was plotted versus chromosome position for all 16 chromosomes for meiotic cells (red, enrichment is upwards) and mitotic cells (blue, enrichment is downwards). Inverted triangles represent the significant binding sites we identified. White dots indicate the position of the centromere on each chromosome. (TIF) [file pgen.1002643.s001.tif]

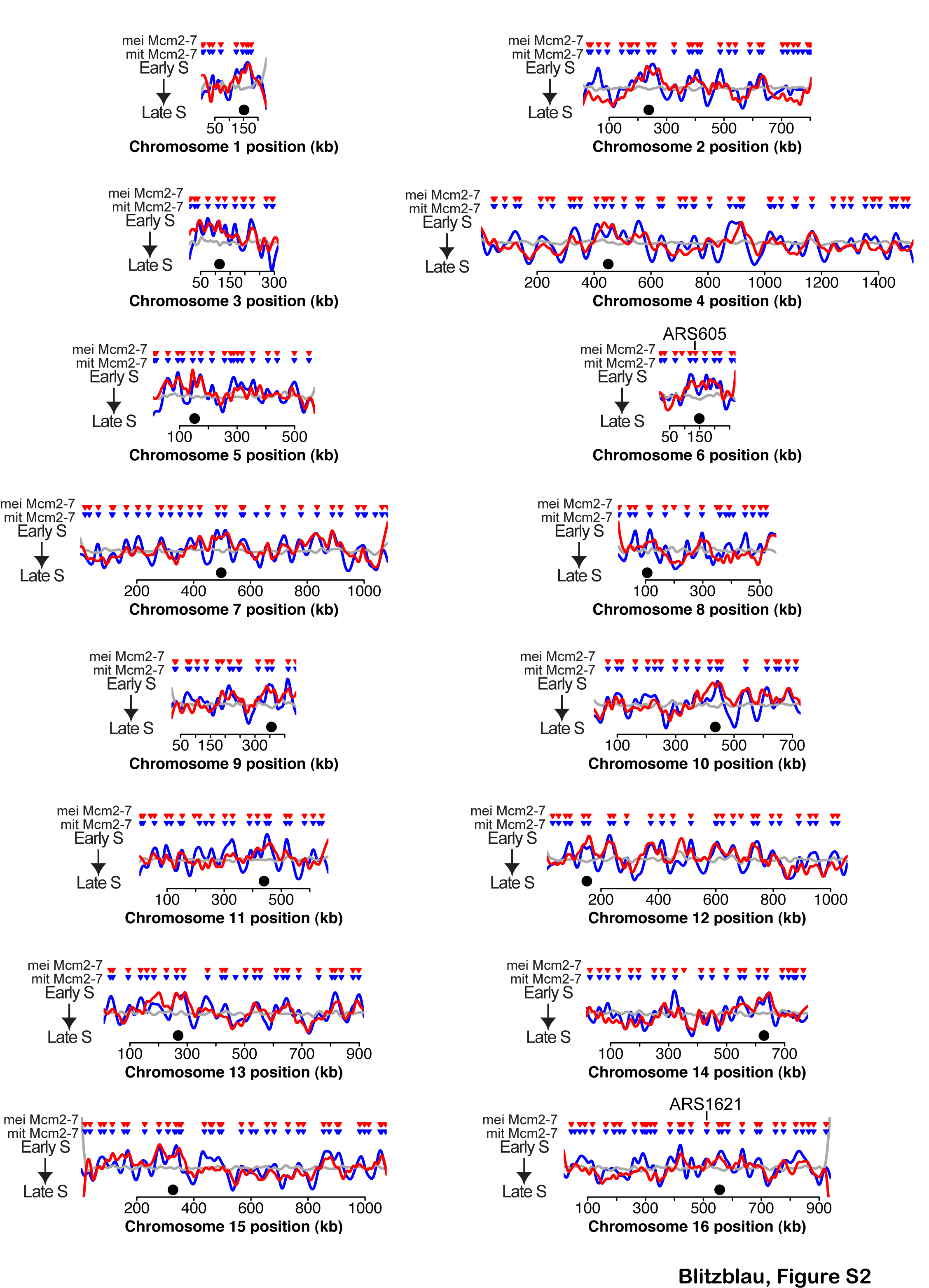

Supplement: Figure S2 — MeiS and mitS replication profiles. The smoothed, predicted replication timing profiles are shown for each of the 16 chromosomes for meiS (red lines), mitS (blue lines) and G1 vs. G1 control hybridization (grey lines). Mcm2-7 binding sites are indicated by inverted triangles for meiotic (red) and mitotic (blue) cells. Black dots indicate the positions of centromeres. (TIF) [file pgen.1002643.s002.tif]

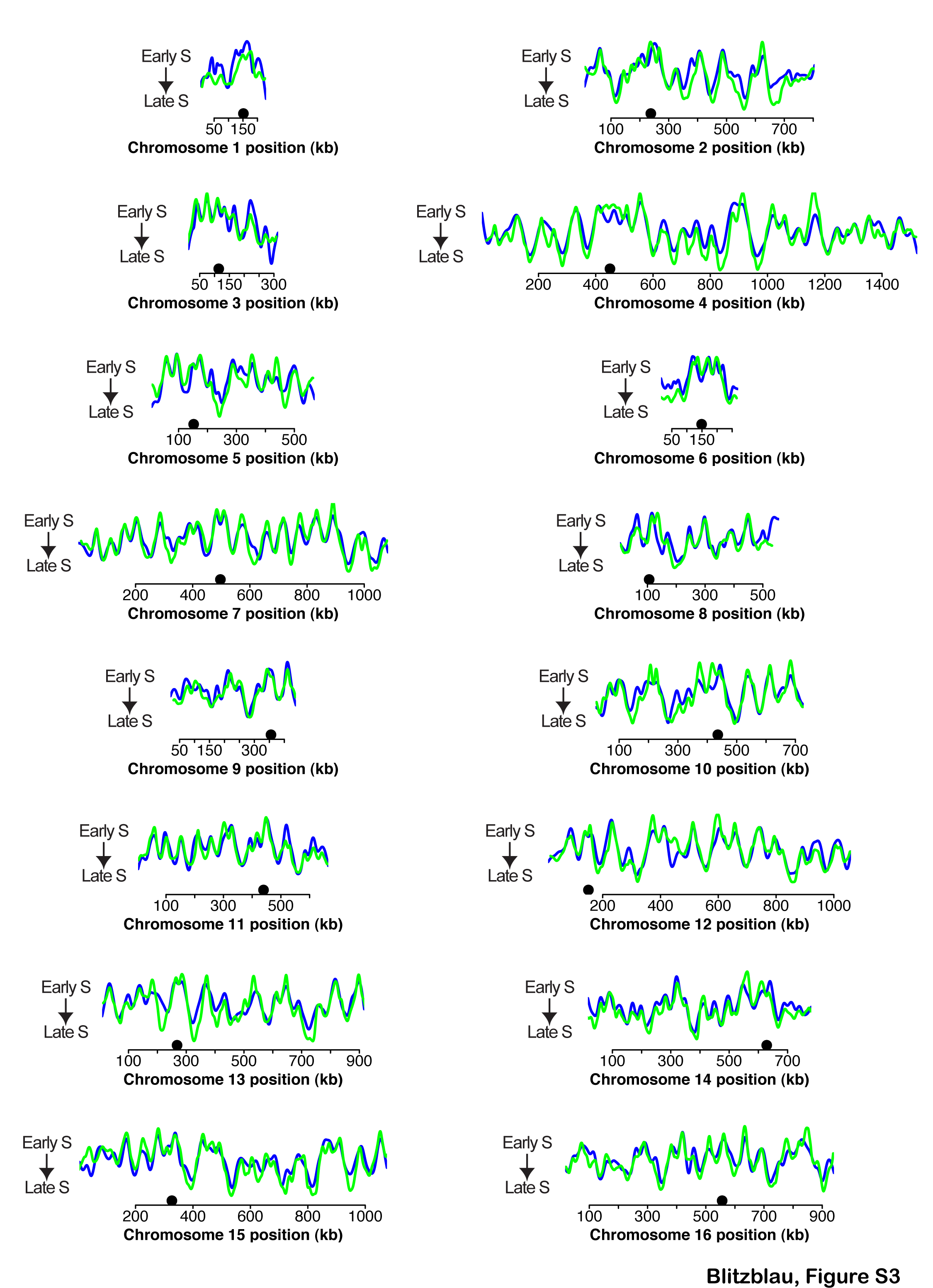

Supplement: Figure S3 — Comparison of mitS replication profiles for 2 yeast strains. MitS replication profiles for all chromosomes are shown for the current study in SK1 (blue lines) and for W303 (green lines) from Yabuki and colleagues [9]. Black dots indicate the positions of centromeres. (TIF) [file pgen.1002643.s003.tif]

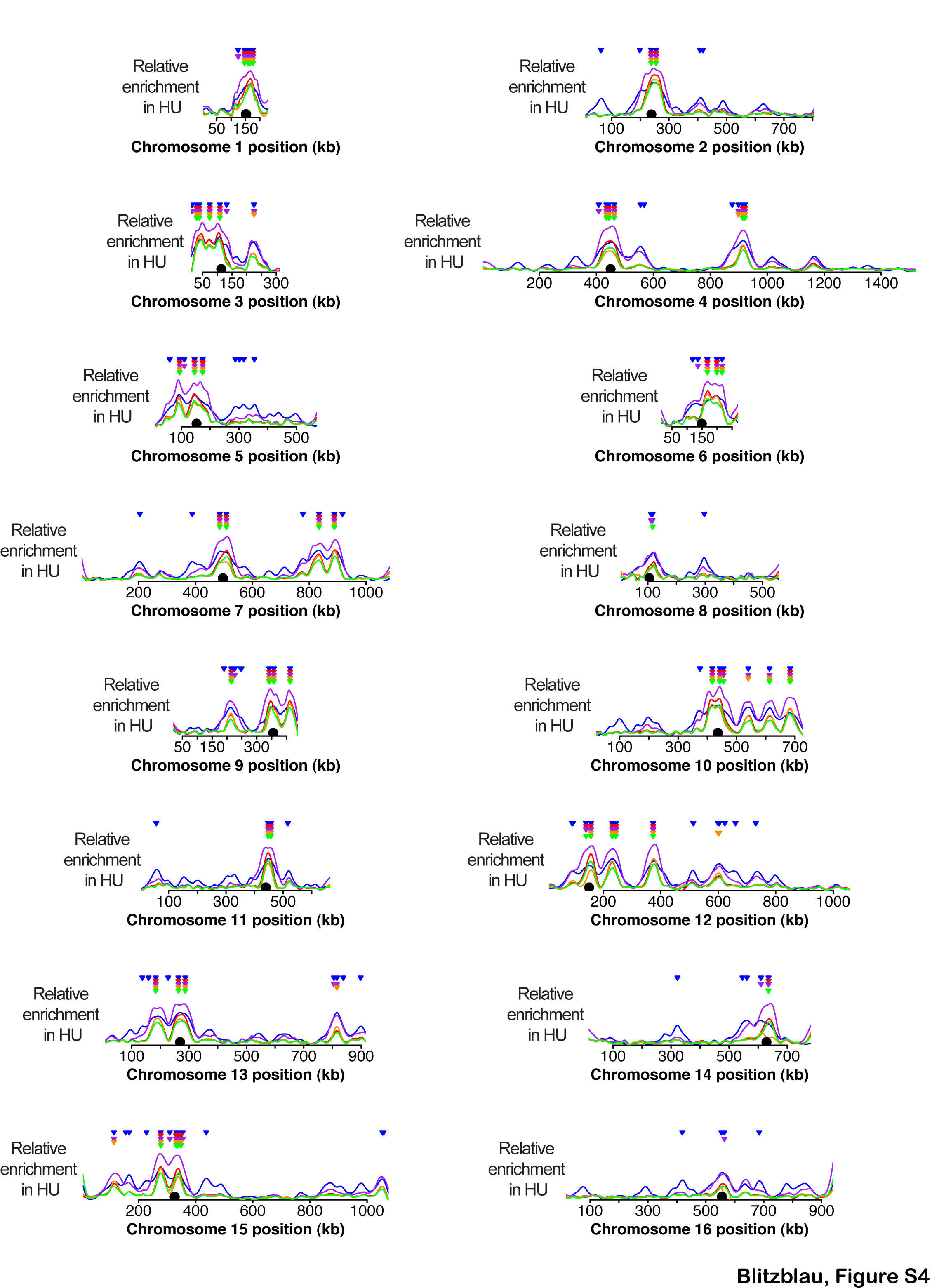

Supplement: Figure S4 — HU replication profiles. The extent of replication after 4 hours in HU is plotted for all 16 chromosomes for wild-type cells (H574) in YPD+200 mM HU (blue lines), SPO+20 mM HU for wild-type cells (H574, red lines), sml1Δ cells (H4898, purple lines), rec8Δ (H5187, orange lines) and spo11Δ (H5184, green lines). Inverted arrowheads denote origins that are replicated in each strain of the corresponding color. Black dots indicate the positions of centromeres. (TIF) [file pgen.1002643.s004.tif]

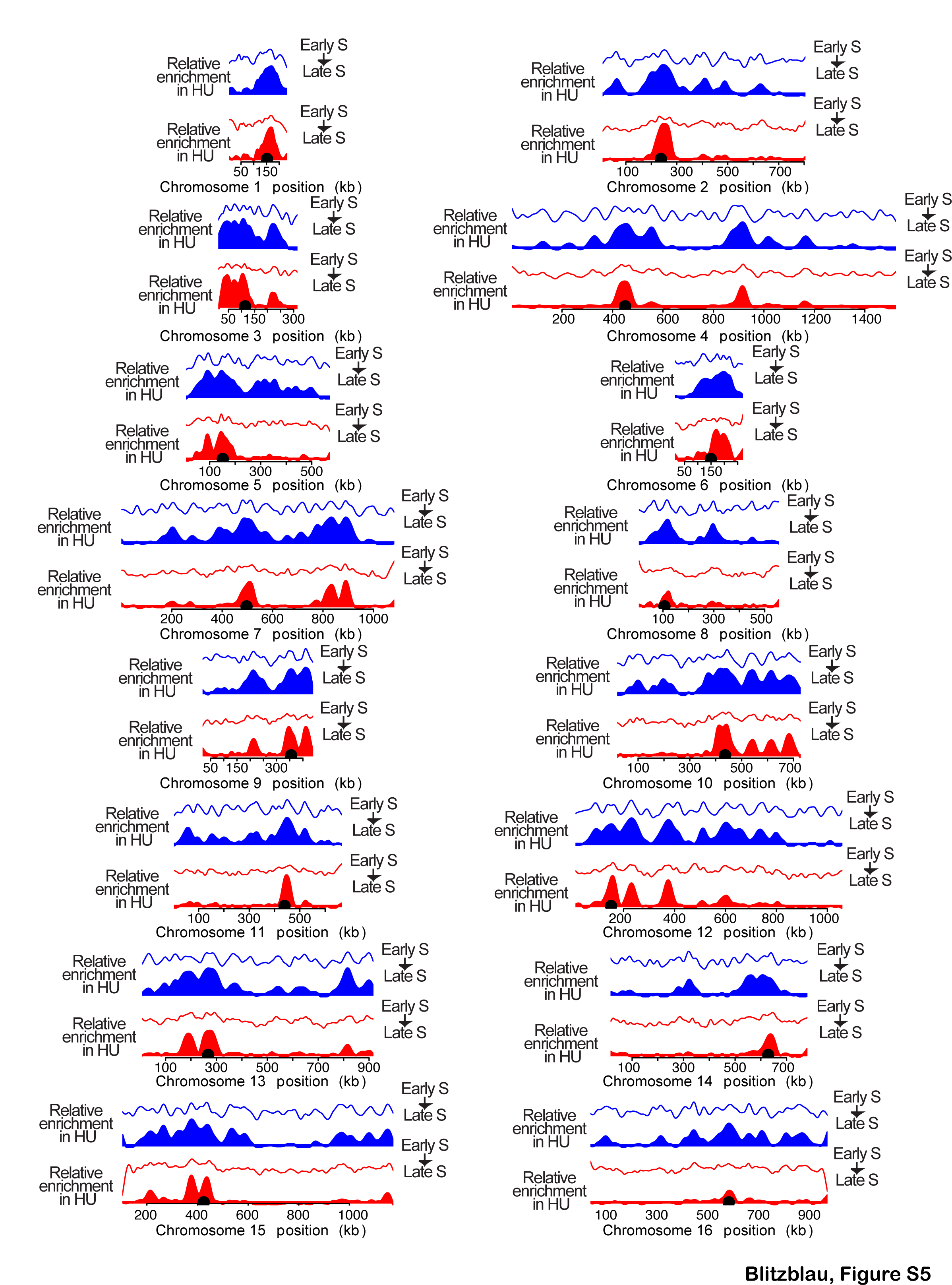

Supplement: Figure S5 — Comparison of timing and HU profiles. The extent of replication after 4 hours in HU is plotted for all 16 chromosomes for wild-type cells (H574) in YPD+200 mM HU (blue histogram) and SPO+20 mM HU for wild-type cells (H574, red histogram). Above each histogram the corresponding S phase replication timing profile is plotted in blue for mitS and red for meiS. Black dots indicate the positions of centromeres. (TIF) [file pgen.1002643.s005.tif]

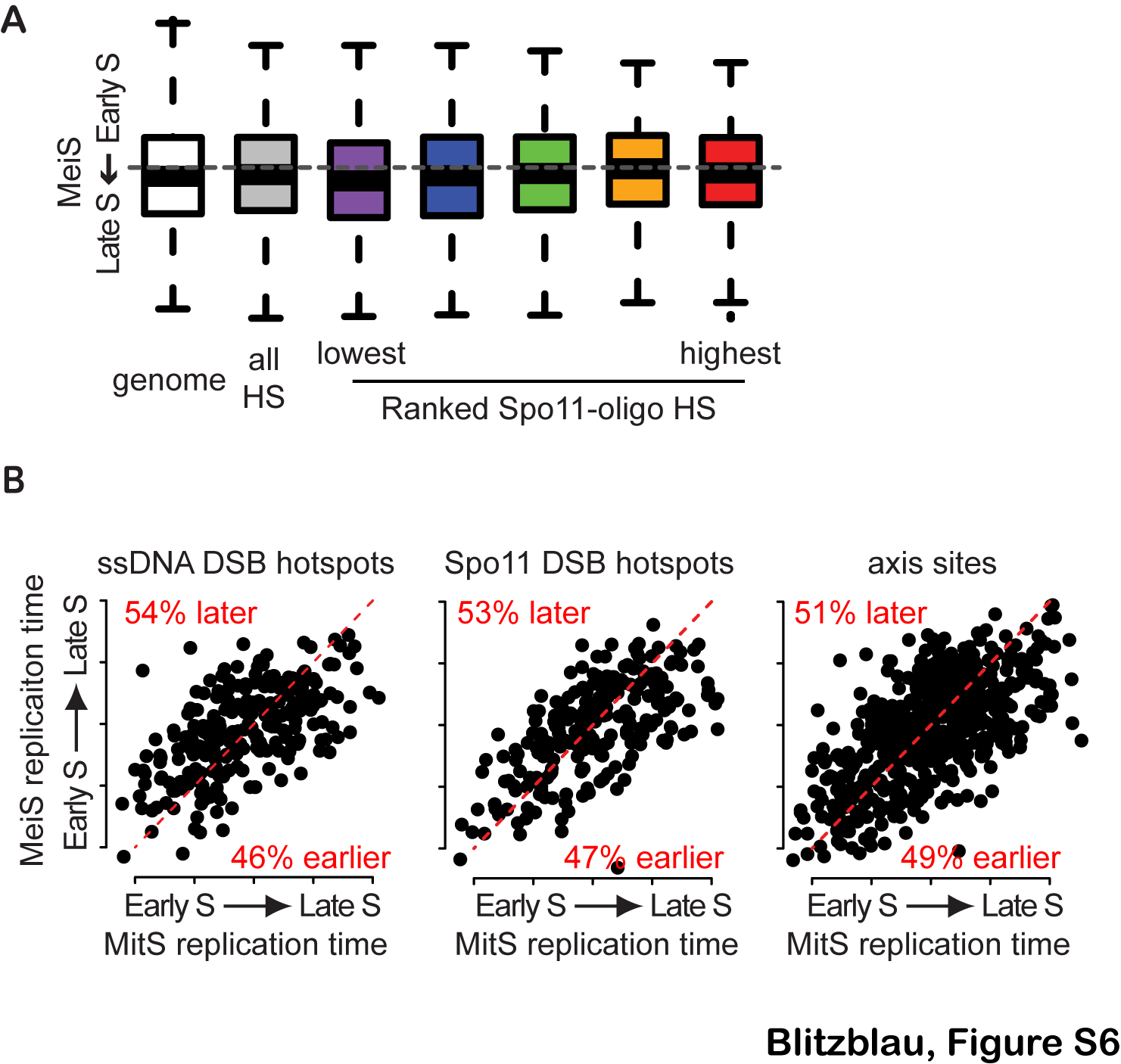

Supplement: Figure S6 — DSB hotspot replication time. (A) The distribution of relative replication time for HSs mapped by Spo11-oligo recovery are shown for all 3434 non-telomeric HSs (grey box), and the 3434 HSs ranked in quintiles from lowest break number (purple) to highest break number (red). The timing distribution for the whole genome is shown as a white box. (B) The meiS replication time of DSB HSs mapped by ssDNA enrichment (left panel) or Spo11 genome-wide location analysis (center panel), or axis association sites (right panel) are plotted as a function of their time of replication in mitS. Red line indicates the predicted trend if relative replication time were identical in meiS and mitS. (TIF) [file pgen.1002643.s006.tif]

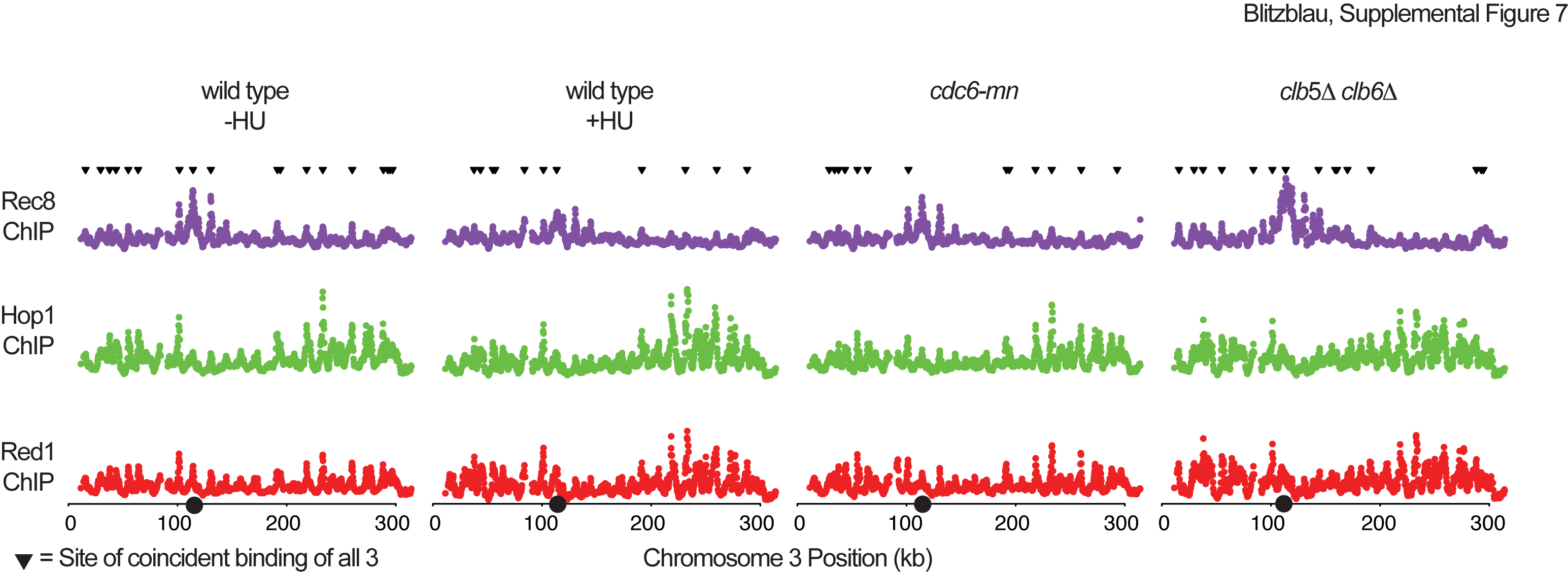

Supplement: Figure S7 — Axis sites. Genome-wide localization analysis was performed for Rec8 (shown in purple), in wild-type cells with and without HU (H4471) cdc6-mn cells (H5491) and clb5Δ clb6Δ cells (H6495). Hop1 localization analysis is shown in green for wild-type cells without HU (H119, [67]), with HU (H4471), cdc6-mn cells (H154) and clb5Δ clb6Δ cells (H2017). Red1 localization analysis is shown in red for wild-type cells without HU (H119), with HU (H4471), cdc6-mn cells (H154) and clb5Δ clb6Δ cells (H2017). The enrichment of immunoprecipitated over input DNA is plotted for chromosome III. Sites that showed significant coincident binding for Rec8, Hop1 and Red1 are indicated by inverted black triangles above the plots. Black dots indicate the positions of the centromere. (TIF) [file pgen.1002643.s007.tif]

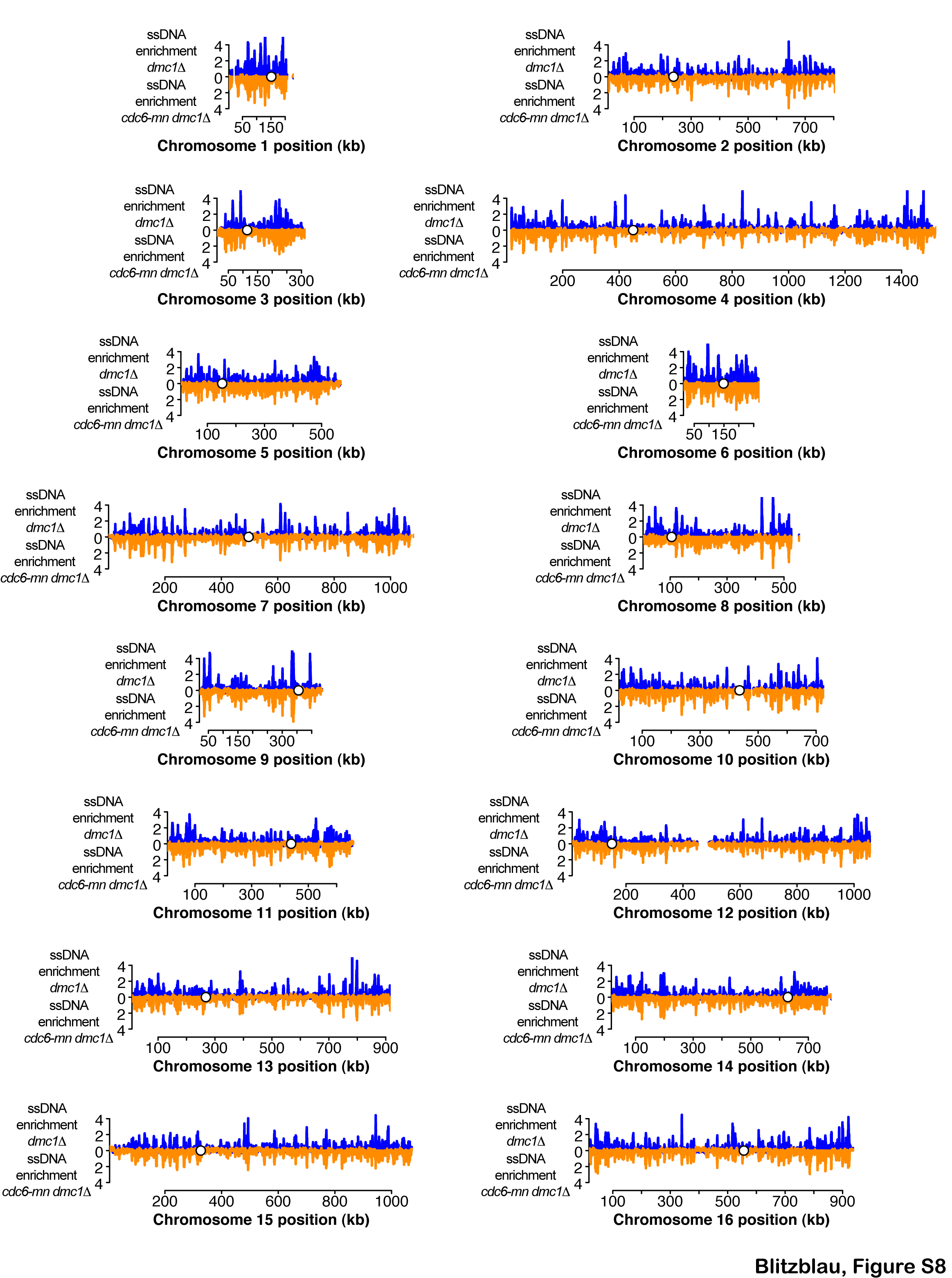

Supplement: Figure S8 — ssDNA enrichment profiles. The ssDNA enrichment profiles for dmc1Δ cells (blue, [67]), and dmc1Δ cdc6-mn cells (H1584, orange, enrichment downwards) were plotted with respect to position for all 16 yeast chromosomes. Black dots indicate the positions of centromeres. (TIF) [file pgen.1002643.s008.tif]
