# Supplementary material for: Separation of DNA Replication from the Assembly of Break-Competent Meiotic Chromosomes
Source: PLoS Genet. 2012 May 17;8(5):e1002643. doi: 10.1371/journal.pgen.1002643 (PMC3355065; doi:10.1371/journal.pgen.1002643)
Supplement: Table S4 — Strains used in this study. (DOC) [file pgen.1002643.s012.doc]

**Blitzblau et al. Table S1.** Genotypes of yeast strains used in this study.

| **Strain No.** | **Relevant genotype** | **Reference** |
| --- | --- | --- |
| A4224 | MATalpha, ho::LYS2, lys2, HIS4, leu2::hisG, trp1::hisG, ura3,  MATa, ho::LYS2, lys2, his4X, leu2::hisG, TRP1, ura3,  cln3Δ::LEU2  cln3Δ::LEU2 | A. Amon |
| SB1505 | MATa, ho::LYS2, lys2, ura3, leu2::hisG. his3::hisG, trp1::hisG, flo8Δ::KanMX | This study |
| A4841 | MATa, ho::LYS2, lys2, ura3, leu2::hisG, his3::hisG, trp1::hisG | A. Amon |
| KBY518 | Mata, ho::hisG, lys2, ura3, leu2::hisG, trp1::hisGDGA,  Matalpha, ho::hisG, lys2, ura3, leu2::hisG, trp1::hisGDGA, his3-11, his3-15, ime2-as1-myc::TRP1(M146G)  his3-11, his3-15, ime2-as1-myc::TRP1(M146G) | Benjamin et al., 2003 [1] |
| H574 | MATalpha, ho::LYS2, lys2, leu2::hisG, his4X  MATa, ho::LYS2, lys2, LEU2, HIS4 | This study |
| H4912 | MATa, ho::LYS2, lys2, ura3, leu2::hisG, his3::hisG, TRP1,  MATalpha, ho::LYS2, lys2, URA3, LEU2, HIS3, trp1::hisG,  sml1Δ::KanMX  sml1Δ::KanMX | This study |
| H5184 | MATa, ho::LYS2, lys2, LEU2, ura3, trp1::hisG,  MATalpha, ho::LYS2, lys2, leu2::hisG, URA3, trp1::hisG  his3::hisG, spo11::TRP1  HIS3, spo11::TRP1 | This study |
| H5187 | MATa, ho::LYS2, lys2, his3::hisG, URA3, leu2::hisG,  MATalpha, ho::LYS2, lys2, his3::hisG, ura3, LEU2,  TRP, rec8::HIS3MX6  trp1::hisG, rec8::HIS3MX6 | This study |
| H154  = A10912 | MATa, ho::LYS2, lys2, ura3, leu2::hisG, his3::hisG,  MATalpha, ho::LYS2, lys2, ura3, leu2::hisG, HIS3,  cdc6::KanMX6::Pscc1: CDC6  cdc6::KanMX6::Pscc1: CDC6  his4X::LEU2-(Bam)-URA3, arg4-Nsp  his4B::LEU2, arg4-Bgl II | Hochwagen et al., 2005 [2] |
| NKY1551 | MATa, ho::LYS2, lys2, ura3, leu2::hisG,  MATalpha, ho::LYS2, lys2, ura3, leu2::hisG,  his4B::LEU2, arg4-Bgl II  his4X::LEU2 (Bam)-URA3, arg4-Nsp | Storlazzi,[3] 1995 |
| H4471 | MATa, ho::LYS2, lys2, ura3, leu2::hisG,  MATalpha, ho::LYS2, lys2, ura3, leu2::hisG,  his4B::LEU2, arg4-Bgl II, REC8::HA3::URA3  his4B::LEU2, arg4-Bgl II, REC8::HA3::URA3 | This study |
| H5491 | MATa, ho::LYS2, lys2, ura3, leu2::hisG, trp1::hisG, HIS3(?),  MATalpha, ho::LYS2, lys2, ura3, leu2::hisG, TRP1, HIS3(?),  his4B::LEU2, cdc6::KanMX6::Pscc1: CDC6, arg4-Bgl II  his4B::LEU2, cdc6::KanMX6::Pscc1: CDC6, REC8::HA3::URA3  REC8::HA3::URA3 | This study |
| H2017 | MATa, ho::LYS2, lys2, ura3, leu2::hisG, HIS3,  MATalpha, ho::LYS2, lys2, ura3, leu2::hisG, his3::hisG,  clb5Δ::KanMX6, clb6Δ::TRP1  clb5Δ::KanMX6, clb6Δ::TRP1  his4X::LEU2-(Bam)-URA3, arg4-Nsp  his4B::LEU2, arg4-Bgl II | This study |
| H6495  =A16113 | MATa, ho::LYS2, lys2, leu2::hisG, ura3, HIS  MATalpha, ho::LYS2, lys2, leu2::hisG, ura3, his3::hisG  clb5Δ::KanMX6, clb6Δ::TRP1, REC8-3HA::URA3  clb5Δ::KanMX6, clb6Δ::TRP1, REC8-3HA::URA3 | Brar et al., 2009 [4] |
| H118  =NKY1455 | MATa, ho::LYS2, lys2, leu2::hisG, ura3, his4X::LEU2-URA3, MATalpha, ho::LYS2, lys2, leu2::hisG, ura3, his4B::LEU2,  arg4-nsp, dmc1Δ::ARG4  arg4-Bgl2, dmc1Δ::ARG4 | Bishop et al., 1992 [5] |
| H1584 | MATa, ho::LYS2, ura3, leu2::hisG, his3::hisG, trp1::hisG,  MATalpha, ho::LYS2, ura3, leu2::hisG, his3::hisG, trp1::hisG,  cdc6::KanMX6::P_scc1_: CDC6, dmc1Δ::HIS3  cdc6::KanMX6::P_scc1_: CDC6, dmc1Δ::HIS3, fpr3Δ::TRP1 | This study |
| H4534 | MATalpha, ho::LYS2, lys2, leu2::hisG, his3::hisG, ura3,  MATalpha, ho::LYS2, lys2, leu2::hisG, his3::hisG(?), ura3, TRP1, his4X::LEU2-URA3, dmc1Δ::ARG4,  trp1::hisG, his4B::LEU2, dmc1Δ::ARG4,  cdc6::KanMX6::pSCC1: CDC6  cdc6::KanMX6::pSCC1: CDC6 | This study |

# References

# 1. Benjamin KR, Zhang C, Shokat KM, Herskowitz I (2003) Control of landmark events in meiosis by the CDK Cdc28 and the meiosis-specific kinase Ime2. Genes Dev 17: 1524-1539.

# 2. Hochwagen A, Tham WH, Brar GA, Amon A (2005) The FK506 binding protein Fpr3 counteracts protein phosphatase 1 to maintain meiotic recombination checkpoint activity. Cell 122: 861-873.

# 3. Storlazzi A, Xu L, Cao L, Kleckner N (1995) Crossover and noncrossover recombination during meiosis: timing and pathway relationships. Proc Natl Acad Sci U S A 92: 8512-8516.

# 4. Brar GA, Hochwagen A, Ee LS, Amon A (2009) The multiple roles of cohesin in meiotic chromosome morphogenesis and pairing. Mol Biol Cell 20: 1030-1047.

# 5. Bishop DK, Park D, Xu L, Kleckner N (1992) DMC1: a meiosis-specific yeast homolog of E. coli recA required for recombination, synaptonemal complex formation, and cell cycle progression. Cell 69: 439-456.
